# Scale Invariant Disordered Nanotopography Promotes Hippocampal Neuron Development and Maturation with Involvement of Mechanotransductive Pathways

**DOI:** 10.3389/fncel.2016.00267

**Published:** 2016-11-18

**Authors:** Carsten Schulte, Maddalena Ripamonti, Elisa Maffioli, Martino A. Cappelluti, Simona Nonnis, Luca Puricelli, Jacopo Lamanna, Claudio Piazzoni, Alessandro Podestà, Cristina Lenardi, Gabriella Tedeschi, Antonio Malgaroli, Paolo Milani

**Affiliations:** ^1^Dipartimento di Fisica, Centro Interdisciplinare Materiali e Interfacce Nanostrutturate, Università degli Studi di MilanoMilan, Italy; ^2^Fondazione FilareteMilan, Italy; ^3^Neurobiology of Learning Unit, Division of Neuroscience, Scientific Institute San Raffaele, Università Vita-Salute San RaffaeleMilan, Italy; ^4^Dipartimento di Medicina Veterinaria, Università degli Studi di MilanoMilan, Italy; ^5^SEMM - European School of Molecular MedicineMilan, Italy

**Keywords:** neuronal differentiation, neuronal network maturation, biomaterial, mechanotransduction, proteomics, synaptic activity, integrin adhesion complex, neuronal cell adhesion molecules

## Abstract

The identification of biomaterials which promote neuronal maturation up to the generation of integrated neural circuits is fundamental for modern neuroscience. The development of neural circuits arises from complex maturative processes regulated by poorly understood signaling events, often guided by the extracellular matrix (ECM). Here we report that nanostructured zirconia surfaces, produced by supersonic cluster beam deposition of zirconia nanoparticles and characterized by ECM-like nanotopographical features, can direct the maturation of neural networks. Hippocampal neurons cultured on such cluster-assembled surfaces displayed enhanced differentiation paralleled by functional changes. The latter was demonstrated by single-cell electrophysiology showing earlier action potential generation and increased spontaneous postsynaptic currents compared to the neurons grown on the featureless unnaturally flat standard control surfaces. Label-free shotgun proteomics broadly confirmed the functional changes and suggests furthermore a vast impact of the neuron/nanotopography interaction on mechanotransductive machinery components, known to control physiological *in vivo* ECM-regulated axon guidance and synaptic plasticity. Our results indicate a potential of cluster-assembled zirconia nanotopography exploitable for the creation of efficient neural tissue interfaces and cell culture devices promoting neurogenic events, but also for unveiling mechanotransductive aspects of neuronal development and maturation.

## Introduction

The ability of engineered biomaterials to guide and control cell biological responses hold a great promise for applications in versatile biomedical contexts, e.g., cell replacement therapies or tissue engineering in regenerative medicine (Hench and Polak, 2002; Lutolf et al., 2009; Mendes, 2013; Dalby et al., 2014; Murphy et al., 2014). In the context of the nervous system, due to the limited intrinsic regenerative capacity of most neuronal cells, many different biomaterials have been screened for their capacity to promote the recapitulation of neurogenic processes and the induction of neuronal maturation necessary for the formation of fully functional synaptic circuits. Such biomaterials would be quite interesting for the advancement of neural circuits or interfaces (Kotov et al., 2009; Franze et al., 2013; Fattahi et al., 2014) and could give an important contribution to the generation of *in vitro* neurodegenerative disease models (Sandoe and Eggan, 2013) or the regeneration/substitution of damaged neurons (Abematsu et al., 2010; Lu et al., 2012; Grealish et al., 2014; Tong et al., 2015).

Although the underlying processes which regulate neuronal differentiation are not fully understood due to their complexity, neuroinductive protocols to obtain mature neurons from adequate stem cell systems have been realized. Existing protocols are based on biochemical and genetic approaches, targeting individual known key players by appropriate growth factors/reagents and/or the induced expression of specific transcription factors (Conti and Cattaneo, 2010; Sandoe and Eggan, 2013; Amamoto and Arlotta, 2014; Maury et al., 2015). However, these protocols are quite delicate, time-consuming and in addition their efficiency is still low. Therefore, solutions to speed up the procedures and to improve the efficiency are under intense search (Sandoe and Eggan, 2013).

The combination of the above mentioned molecular neuroinduction strategies with additional adequate biophysical stimuli provided by synthetic biomaterial substrates could reach this goal (Discher et al., 2009; Mammadov et al., 2013; Tong et al., 2015). The capacity of biomaterials to modulate cellular functions relies on the cellular competence for mechanotransduction; i.e., the perception of microenvironmental biophysical signals (rigidity and nanotopography) and the subsequent conversion into corresponding cellular responses via mechanosensitive cell components (Wang et al., 2009; Dalby et al., 2014; Murphy et al., 2014; Chen et al., 2015). The phenomen of cellular biomechanics, in particular its involvement in neurogenesis and neuronal development, has attracted considerable interest in the last years (Tyler, 2012; Franze et al., 2013; Kerstein et al., 2015).

Many attempts try to exploit the potential of substrate rigidity modulation in fostering neuronal differentiation (Franze et al., 2013; Mammadov et al., 2013). For neural or pluripotent stem cells it was demonstrated that neural commitment can be enhanced by using soft biomaterials as cell culture substrate (Saha et al., 2008; Keung et al., 2013; Mammadov et al., 2013; Musah et al., 2014). In two recent studies electrophysiological measurements also confirmed the proper functionality of the obtained neurons (Musah et al., 2014; Sun et al., 2014). The regulation of the neuronal differentiation/maturation-promoting effects of soft substrates was associated with the protein YAP (Musah et al., 2014; Sun et al., 2014), an important mediator in mechanotransduction (Halder et al., 2012).

Another strategy in biomaterial engineering is based on mimicking topographical features found in the extracellular matrix (ECM) by the fabrication of nanostructured surfaces (Kim et al., 2012; Gasiorowski et al., 2013; Mendes, 2013; Dalby et al., 2014; Murphy et al., 2014; Chen et al., 2015). The importance of neuron/ECM interaction for neurogenic events is well-documented (Pizzorusso et al., 2002; de Curtis, 2007; Dityatev et al., 2010; Myers et al., 2011; Kerstein et al., 2015). Neural circuit development critically depends on the generation of well-defined dendritic and axonal structures and their eventual reciprocal interactions leading to functional synaptic junctions (Benson et al., 2001; Graf et al., 2004; Nam and Chen, 2005; Sara et al., 2005). The appropriate match between the two elements of a future synapses is mediated by members of the cadherin, immunoglobulin, and integrin families. These developmental processes are largely controlled by extracellular cues which can be diffusible but often they are bound to cell membranes or are part of the ECM providing attractive, repulsive, or retaining signals like e.g., in the perineuronal nets. Especially the outgrowth/guidance of axons and the synaptic plasticity are modulated by a spatiotemporally dynamic interaction with the substrate (Benson et al., 2001; Pizzorusso et al., 2002; Craig et al., 2006; de Curtis, 2007; Dityatev et al., 2010; Myers et al., 2011; Vitriol and Zheng, 2012; Geissler et al., 2013; Bikbaev et al., 2015; Kerstein et al., 2015). For the exploration of the microenvironment integrin-mediated point contacts play an essential role by linking the ECM to the neuronal actin cytoskeleton which enables force generation and mechanotransduction. The mechanotransductive signal processing is realized by the force-dependent recruitment of an elaborated network of structural, cytoskeletal and signaling components creating the integrin adhesion complexes (IAC) (de Curtis, 2007; Dityatev et al., 2010; Betz et al., 2011; Myers et al., 2011; Kerstein et al., 2015; Nichol et al., 2016).

Neuronal cells are known to be competent in sensing precisely topographical surface differences and to respond to this kind of nanoscale information (Brunetti et al., 2010; Chua J. S. et al., 2014). Indeed, it has been demonstrated that the polarization of neurite/axon outgrowth can be controlled by topographical cues (Hoffman-Kim et al., 2010; Ferrari et al., 2011). Several studies suggest furthermore a positive contribution of biomaterials with appropriate nanotopographical substrate features to neuronal differentiation in diverse neuronal or stem cell types (Foley et al., 2005; Cellot et al., 2009; Christopherson et al., 2009; Malarkey et al., 2009; Lee et al., 2010; Wu et al., 2010; Fabbro et al., 2012; Tamplenizza et al., 2013; Kulangara et al., 2014; Yang et al., 2014, 2016; Schulte et al., 2016) and recent data propose a prominent involvement of IAC (Yang et al., 2014, 2016; Schulte et al., 2016). However, a more detailed molecular insight into the underlying mechanotransductive processes and the determination of the key players regulating nanotopography-mediated impact on neurogenic events is needed.

In this framework, we have recently analyzed the specific effects induced in the neuron-like PC12 cell line by the interaction with nanostructured zirconia surfaces (Schulte et al., 2016) fabricated by supersonic cluster beam deposition (SCBD) of zirconia nanoparticles (Wegner et al., 2006). We found that the nanotopographical features of these cluster-assembled surfaces can manipulate the IAC nanoarchitecture, dynamics and composition which leads to mechanotransductive signaling events. These data suggested a potential of this biomaterial as modulator of neuronal differentiation (Schulte et al., 2016). In this present work, we have used primary hippocampal neurons, a standard model to study neurogenesis and the functional synaptic network integration (Raineteau et al., 2004; Cheyne et al., 2011), to evaluate the potential outcomes of nanotopographical features provided by nanostructured zirconia surfaces on the development of neuronal morphology, synaptogenesis, and network maturation.

## Materials and methods

### Fabrication of nanostructured zirconia surfaces by supersonic cluster beam deposition

The nanostructured surfaces were fabricated by supersonic cluster beam deposition (SCBD) as described elsewhere in detail (Wegner et al., 2006). Summarizing, clusters are formed by ablation and thermolization of a metal rod by argon plasma (ignited by pulsed electric discharges). The cluster/plasma mixture expands through a nozzle into a vacuum and is aerodynamically focused to a supersonic beam. This focused beam of nanoparticles impinges on the substrate placed into the beam. Thereby a nanostructured film of defined thickness and roughness can be grown. Standard glass and flat zirconia surfaces (the latter produced by e-Beam evaporation) were used as references.

### Characterization of substrate surface morphology by atomic force microscopy

The surface morphology of cluster-assembled zirconia films and the flat glass and zirconia substrates were characterized by Atomic Force Microscopy (AFM) operated in Tapping Mode in air, using a Multimode AFM equipped with a Nanoscope IV controller (Bruker, Billerica, Massachusetts, USA). Rigid silicon cantilevers (k≈40 N/m, f_0_≈300 kHz) mounting single crystal silicon tips with nominal radius 5–10 nm have been used. For each sample, 2–3 images with dimensions 2 × 1 μm were acquired on macroscopically separated regions, with scan rate in the range 0.4–0.8 Hz and sampling resolution of 2048 × 512 points. The images were flattened by line-by-line subtraction of first and second order polynomials in order to remove artifacts due to sample tilt and scanner bow. From flattened AFM images root-mean-square surface roughness R_q_ was calculated as the standard deviation of surface heights. The associated error δ_tot_ was evaluated by summing in quadrature the standard deviation of the mean $\sigma_{\text{mean}}=\frac{\sigma }{\sqrt{\text{N}}}$ with σ and N representing respectively the standard deviation and the number of acquired images for each sample, and an effective relative error given by σ_instrum_ = 5.5 % accounting for piezo calibration uncertainty and artifacts related to tip convolution issues. The global error was thus evaluated as $\sigma_{\text{tot}}\;=\;\sqrt{\sigma_{\text{instrum}}^{2}{\text{R}}_{q}^{2}+\sigma_{\text{mean}}^{2}}$. The same experimental protocol was applied to all the different analyzed surfaces (Control (glass coverslips), flat-Zr, ns-Zr15, ns-Zr25), aimed at reproducing the sequence of treatments typically applied to substrates before culturing the hippocampal neurons. First the bare substrates were characterized by AFM, followed by an overnight incubation with 1 μg/ml polyornithine (Sigma-Aldrich, St. Louis, Missouri, USA) in PBS and a second set of AFM measurements; the final set of measurements was performed after incubation with diluted matrigel (1:50 diluted stock solution, 30 min.) (Becton Dickinson, Franklin Lakes, New Jersey, USA) and culture medium (15 min., composition see next section). Before each set of measurements, the samples were gently rinsed with Milli-Q water in order to remove the excess or loosely bound material, and then gently dried with pure nitrogen stream.

### Postnatal hippocampal neuronal cultures

Postnatal hippocampal cultures were prepared as previously described (Malgaroli and Tsien, 1992). Research and animal care procedures were performed as approved by the Institutional Animal Care and Use Committee for Good Animal Experimentation of the Scientific Institute San Raffaele according the code of practice for the care and use of animals for scientific purposes of the Italian Ministero della Salute (IACUC number: 576).

In brief, postnatal (P2 pups) were decapitated, after which the hippocampus was separated in cold dissociation medium [1 L of dissociation medium: 350 mg NaHCO_3_, 2.38 g HEPES, 6 g glucose, 38 mg kynurenic acid (R&D System, Tocris, Minneapolis, Minnesota, USA), 300 mg BSA, 1.444 g magnesium sulfate, 5 mg gentamycin, 1 L Hank's salt solution, pH 7.3] and enzymatic digestion of the hippocampal tissue was run 100 ml digestion medium: 800 mg NaCl, 37 mg KCl, 99 mg NaHPO_4_, 600 mg HEPES, 35 mg NaHCO_3_, 3.8 mg kynurenic acid, pH 7.4, 3 mg/ml trypsin, 1 mg/ml DNAaseI (Merck Millipore, Calbiochem, Billerica, Massachusetts, USA), 5 min, room temperature. The cells were mechanically dissociated by a serological pipette in dissociation medium supplemented with 1 mg/ml DNAaseI (Merck Millipore, Calbiochem). An equal volume of isolated neurons was plated on control, flat, and nanostructured zirconia surfaces. Prior to plating the cells (~3 ∗ 10^5^ cells/cm^2^), each surface was coated with 1 μg/ml polyornitine overnight and then Matrigel® (Becton Dickinson) (20 μl of 1:50 diluted stock solution) was added to the coverslips 30 min before cell seeding. Cells were grown in the following cell culture conditions: 37°C, 5% CO_2_ and maintained in a custom culture media 1 L of culture medium: 5% fetal calf serum (Thermo Fisher Scientific, Gibco, Massachusetts, USA), 30 mg insulin, 0.1 mg biotin, 1.5 mg B12 vitamin, 100 mg L-ascorbic acid, 100 mg transferrin, 100 mg Glutamax (Thermo Fisher Scientific, Gibco), 7 g glucose, 3.6 g HEPES in 1 L of MEM (Thermo Fisher Scientific, Gibco). Cells were grown for 3–7 DIV, every 3 days 1/3 of the culture medium volume was replaced with fresh one supplemented with ARA-C (2.5–5 μM), to prevent excessive glial cell proliferation. All reagents to which we did not assign a company were purchased from Sigma Aldrich, St. Louis, Missouri, USA.

### Immunofluorescence imaging

The hippocampal neurons were fixed with 4% PFA/phosphate buffer 120 mM pH 7.4, permeabilized and blocked with 0.4% saponin/1%BSA in phosphate buffer 120 mM pH 7.4. The primary antibody was incubated for at least 1 h at room temperature (or alternatively overnight at 4°C) in humid conditions, the secondary antibody (from Jackson Immuno Research Labs, West Grove, Pennsylvania, USA) at room temperature for maximum 1 h. Sample mounting was performed with FluorSave™ (Merck Millipore, Calbiochem) or ProLong® Gold antifade (Thermo Fisher Scientific, Molecular Probes).

The confocal images were recorded with a confocal microscope (Leica TCS SP5, Leica, Wetzlar, Germany) equipped with built-in Argon Laser and Leica 20x DRY (NA 0,5) and 40x OIL (NA 0,5) objectives (Leica) or laser scanning confocal microscope LSM510 with 63x OIL (NA 1,4) objective (Zeiss).

### Analysis of neuron density and clustering

Random fields were acquired for each condition and the number of neurons [identified by NeuN (antibody from Merck Millipore) expression] in each field of view (always with the same dimension), named Neuron Density, was determined and normalized to the Control 3 DIV condition. For the clusterization analysis, centroids of neurons were analyzed by a Matlab (Mathworks, Natick, Massachusetts, USA) code derived by a “kmeans” iterative algorithm. The xy-distance between centroids was measured by squared Euclidean distance and minimized with respect to this parameter. Only groups of neurons composed by *n* > 2 elements were considered as clusters.

### Neuronal morphology reconstruction

Neurons were transduced with a lentivirus that codifies for an eGFP-VAMP2 in order to visualize axons, dendrites and cell bodies. 4 h after plating the neurons were infected with a final viral titer of ~10^6^ TU/ml by directly diluting the lentiviral suspension into the culture medium. Samples were analyzed by confocal microscopy (Zeiss) and the images edited by Adobe Photoshop software (Adobe Systems, San Jose, California, USA).

### Quantification of the neurite outgrowth

Images of cells immunolabeled with MAP2 (antibody from Cell Signaling, Danvers, Massachusetts, USA; or Sigma-Aldrich) were recorded with a confocal microscope and analyzed using ImageJ (NIH, New York, New York, USA). A macro was exploited which runs different morphological ImageJ plugins allowing an automated neurite/dendrite tracing and measure [for further details see Pool et al. (2008)]. The obtained total neurite length was divided by the number of neurons visible in each image. The data are presented as normalized with respect to the mean of the 3 DIV Control condition due to inter-experiment variability using a primary cell system. To smoothen the variability caused by zonal differences (e.g., regarding neuron density or staining intensity) inside the sample, outliers were removed according to a 2 *SD* threshold.

### Quantification of synaptic density

Immunofluorescence images [MAP2 (antibody from Cell Signaling, Danvers, Massachusetts, USA; or Sigma-Aldrich) and p65 (antibody from Synaptic Systems, Goettingen, Germany) staining] of the cells were acquired with a confocal microscope. The collected images were analyzed using ImageJ (NIH, New York, New York, USA) and following a protocol described by Verstraelen et al. (2014). Summarizing, for each field of view the maximum intensity projections of 20x images of MAP2-labeled cells were examined to determine the surface area occupied by the dendrites and to obtain gross information about the network morphology. The synaptic density was quantified by analyzing maximum intensity projections of both, MAP2 and p65 staining, in 40x images. The p65+ spots, representing presynaptic varicosities, were defined by applying a dimension threshold 0.8–1.3 μm^2^ and successive counting of the single spots with the ImageJ plug-in “Analyze Particles.” The synaptic density was then determined as a ratio between the number of p65+ spots and the MAP2+ area in the same field. All the data are reported as normalized with respect to the mean of the 3 DIV Control condition because of the inter-experiment variability of the absolute numbers due to the primary cell system. To smoothen the variability due to zonal differences (e.g., regarding neuron density or staining intensity) inside the sample, outliers were removed according to a 1.5 *SD* threshold.

### Whole cell recordings

Miniature recordings were run on day 3 and day 7 after plating. During the recordings neurons were superfused with tyrode (1–2 ml/min; 24°C; bubbled with 100% O_2_; containing 119 mM NaCl, 5 mM KCl, 2 mM CaCl_2_, 2 mM MgCl_2_, 25 mM HEPES, and 30 mM D-glucose). For mPScs (minis) recordings, the voltage-gated sodium channel blocker tetrodotoxin (TTX) (Latoxan, Portes-lès-Valence, France) was added to the tyrode solution (TTX; 1 μM). The recording pipette (Tip diameter ≈ 1 μm; resistance R_pipette_ 6–8 MΩ) was filled with intracellular solution (gluconic acid 110 mM, MgCl_2_ 5 mM, NaCl 10 mM, EGTA 0.6 mM, ATP 2 mM, GTP 0.2 mM HEPES 49 mM adjusted to pH 7.2, and 290 mOsm) and connected to a patch-clamp amplifier (Axopatch 200B; R&D Systems, Molecular Devices). In voltage-clamp mode (VC) the potential was held at the zero-current, pipette was lowered to selected cells and a GΩ seal was obtained applying slight suction, after holding cell potential to −70 mV full access to cell was obtained by suction-induced opening of plasma membrane. The holding potential was kept to −70 mV for all the recording epoch. The membrane and series resistances were constantly monitored by applying 2–5 mV depolarising pulses. The action potential firing was achieved in current clamp mode by injecting increasing steps of current. The recordings which did not show a stable input and series resistance were discarded. Traces were filtered at 2–5 kHz and acquired using a 16-bit analog-to-digital interface (20 KHz sampling rate, HEKA ITC-18; HEKA Elektronik, Holliston, Massachusetts, USA) controlled by a Labwiew acquisition software developed in house.

All reagents with no assigned company were purchased from Sigma Aldrich, St. Louis, Missouri, USA.

### Mini detection algorithm and statistical analysis

Minis were extracted by means of a custom detection algorithm based on wavelet filtering (MATLAB®, MathWorks) as previously described (Lamanna et al., 2015). For statistical analysis Wilcoxon signed rank test was used. Mini amplitude and frequency were averaged on each recording/cell. The error bars are SEM as indicated in the text and in figure legends. Statistical tests were executed using Matlab built-in functions (Mathworks).

### Proteomics

The cells interacted for 3 days with the indicated substrates (in total 4 coverslips with Ø13 mm each, representing 5.3 cm^2^ cumulative substrate area). Then the cells were scratched from the substrates with a cell scraper (TPP, Trasadingen, Switzerland) (on ice) in the presence of icecold PBS supplemented with protease inhibitors (Roche, Basel, Switzerland).

After reduction and derivatization, the proteins were digested with trypsin sequence grade trypsin (Roche) for 16 h at 37°C using a protein:trypsin ratio of 1:50. LC-ESI-MS/MS analysis was performed on a Dionex UltiMate 3000 HPLC System with a PicoFrit ProteoPrep C18 column (200 mm, internal diameter of 75 μm) (New Objective, USA). Gradient: 1% ACN in 0.1% formic acid for 10 min, 1–4% ACN in 0.1% formic acid for 6 min, 4–30% ACN in 0.1% formic acid for 147 min and 30–50% ACN in 0.1% formic for 3 min at a flow rate of 0.3 μl/min. The eluate was electrosprayed into an LTQ Orbitrap Velos (Thermo Fisher Scientific) through a Proxeon nanoelectrospray ion source (Thermo Fisher Scientific). The LTQ-Orbitrap was operated in positive mode in data-dependent acquisition mode to automatically alternate between a full scan (m/z 350–2000) in the Orbitrap (at resolution 60000, AGC target 1000000) and subsequent CID MS/MS in the linear ion trap of the 20 most intense peaks from full scan (normalized collision energy of 35%, 10 ms activation). Isolation window: 3 Da, unassigned charge states: Rejected, charge state 1: Rejected, charge states 2+, 3+, 4+: Not rejected; dynamic exclusion enabled (60 s, exclusion list size: 200). Five technical replicate analyses of each sample were performed. Data acquisition was controlled by Xcalibur 2.0 and Tune 2.4 software (Thermo Fisher Scientific) (Aletti et al., 2016).

The mass spectra were analyzed using MaxQuant software (version 1.3.0.5) (Cox and Mann, 2008). The initial maximum allowed mass deviation was set to 6 ppm for monoisotopic precursor ions and 0.5 Da for MS/MS peaks. The enzyme specificity was set to trypsin, defined as C-terminal to arginine and lysine excluding proline, and a maximum of two missed cleavages were allowed. Carbamidomethylcysteine was set as a fixed modification, N-terminal acetylation, methionine oxidation and serine/threonine/tyrosine phosphorylation as variable modifications. The spectra were searched by the Andromeda search engine against the rat Uniprot sequence database (release 04.07.2014) and the mouse Uniprot sequence database (release 04.07.2014). The reversed sequences of the target database were used as decoy database. Protein identification required at least one unique or razor peptide per protein group. The quantification in MaxQuant was performed using the built-in XIC-based label free quantification (LFQ) algorithm using fast LFQ (Cox and Mann, 2008). The required false positive rate was set to 1% at the peptide and 1% at the protein level against a concatenated target decoy database, and the minimum required peptide length was set to 6 amino acids. Statistical analyses were performed using the Perseus software (version 1.4.0.6, www.biochem.mpg.de/mann/tools/). Only proteins present and quantified in at least 3 out of 5 technical repeats were considered as positively identified in a sample and used for statistical analyses. An ANOVA test (false discovery rate 0.05) was carried out to identify proteins differentially expressed among the three conditions.

We performed the comparison between cells grown on nanostructured zirconia with the roughness R_q_ of 25 nm rms and the flat surfaces; i.e., Control (glass coverslips) and flat-Zr, in order to better understand the effect of the surface nanotopography. Common proteins were considered to be differentially expressed if they were present only in Control, flat-Zr, or the ns-Zr25 or showed a significant *t*-test difference (cut-off at 5% permutation-based False Discovery Rate). These proteins were filtered for further analyses. Proteins known to be due to a contamination of the matrigel were excluded from the analysis.

The differently expressed proteins were clustered according to their functions using the Panther platform (Version 10.0 release date April 25, 2015) (Mi et al., 2013) and filtered for significant Gene Ontology terms: Biological Process (GO-SlimBP) and Pathways using a *p* value < 0.05.

Genuine mitochondrial protein localization was determined by Mitominer, a database of the mitochondrial proteome which integrates protein data from HomoloGene, Gene Ontology, KEGG, OMIM MS/MS, GFP (green fluorescent protein) localization data and targeting sequence predictions. Only proteins with an Integrated Mitochondrial Protein Index (IMPI) ≥ 0.5 were considered true mitochondrial molecules (Smith et al., 2012).

## Results

### Fabrication and characterization of the cluster-assembled nanostructured zirconia surfaces

Nanoengineered surfaces that mimic ECM topographical features have a considerable potential to modify cellular behavior and fate effected by mechanotransduction-dependent processes, but many details remain elusive (Kim et al., 2012; Gasiorowski et al., 2013; Mendes, 2013; Dalby et al., 2014; Murphy et al., 2014; Chen et al., 2015).

**Figure 1:**
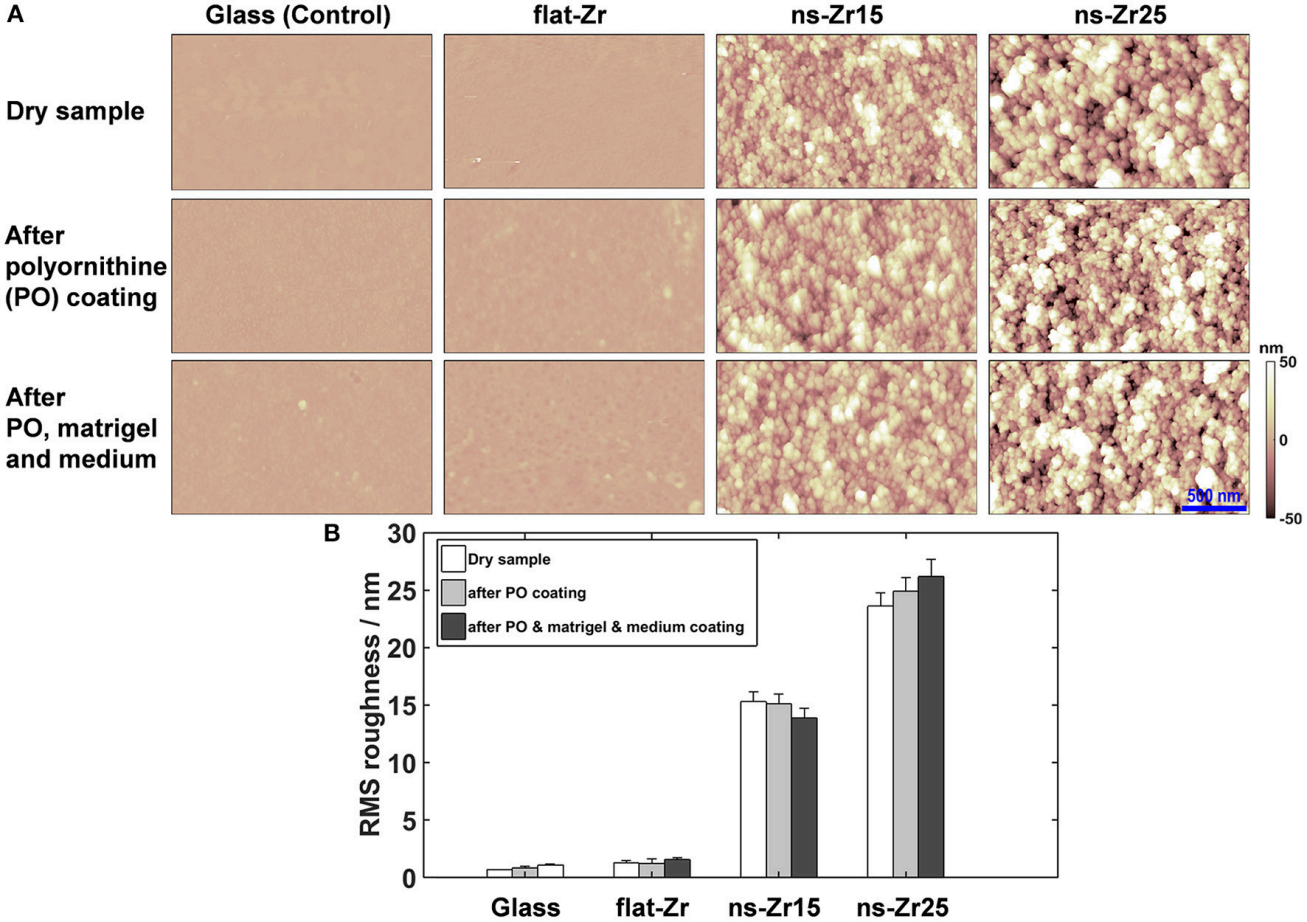
**Surface characterization of the different substrates after polyornithine coating and incubation with diluted matrigel and culture medium. (A)** The images display representative top views of AFM surface characterisations of the diverse indicated substrate conditions glass-control (Control), flat zirconia produced by e-beam evaporation (flat-Zr), nanostructured zirconia produced by SCBD with the roughnesses 15 nm rms (ns-Zr15), respectively 25 nm rms (ns-Zr25) in the dry, original condition (first row), after polyornithine coating (middle row) and matrigel/medium incubation (last row). **(B)** The graph summarizes the quantification of the roughness before and after these different treatments obtained from the AFM images.

In this context, our nanotechnological bottom-up approach is based on the fabrication of nanostructured surfaces by supersonic cluster beam deposition of zirconia nanoparticles obtained with a deposition apparatus equipped with a pulsed microplasma cluster source (Wegner et al., 2006). With the help of this technique it is possible to create reproducible nanostructured films with controllable nanotopographical features (representative examples in Figure 1A) (Wegner et al., 2006; Podestà et al., 2015). Compared to other nanofabrication techniques (especially top-down lithographic approaches) (Mendes, 2013; Chen et al., 2015), SCBD provides the additional advantage to enable the coverage of large macroscopic areas with a defined surface nanotopography. Two different batches of cluster-assembled ZrO_2_ films (ns-Zr) with roughness parameters of R_q_ 15 and 25 nm (ns-Zr25) rms were produced. The surface profiles are characterized by a complex disordered distribution of asperities that at the nanoscale form features comparable in dimensions and spatial organization to the ones found in the ECM (Kim et al., 2012; Gasiorowski et al., 2013). These surface characteristics were the result of the ballistic deposition regime leading to a random hierarchical scale-invariant self-organization of the nanoscale building blocks (nanoclusters) into larger units (Wegner et al., 2006; Podestà et al., 2015).

In a recent publication we showed that these nanostructured surfaces have the capacity to modulate cell adhesion-related parameter, i.e., the IAC nanoarchitecture/dynamics. This is accompanied by a modulation of the cellular nanomechanical properties and promotes neuronal differentiation processes in the neuron-like PC12 cells (Schulte et al., 2016).

In this work we determined whether the potential of the cluster-assembled zirconia surfaces in fostering processes of neuronal differentiation can be verified in a clinically more relevant cell model; primary neurons dissociated from the rat neonatal hippocampus. The standard culturing condition of these cells requires a polyornithine (PO) coating of the glass substrate and the addition of highly diluted matrigel before plating the cells (Malgaroli and Tsien, 1992), this condition served also as canonical cell culture reference (Control). As further control we integrated a flat zirconia surface produced by e-Beam evaporation (flat-Zr). To understand whether the mentioned coating steps compromise the nanotopographical features of the substrates we visualized and characterized the surfaces on the nanoscale by atomic force microscopy (AFM) before and after the different steps of the substrate preparation (Figure 1).

With the exception of the naked glass vs. glass/polyornithine/matrigel/medium (*p* < 0.05, two-tailored *t*-Test), the differences in R_q_ values before and after treatment were not significant (*p* > 0.2–0.5, two-tailored *t*-Test), validating that the surface roughness was not affected by the treatments (Figure 1B). In particular, the characteristic nanotopographical structure of the cluster-assembled surfaces is maintained in the actual experimental condition in which the cells encounter the substrates.

### Effects of the nanotopographical surfaces on neuronal adhesion, viability, morphology, and neurite outgrowth

**Figure 2:**
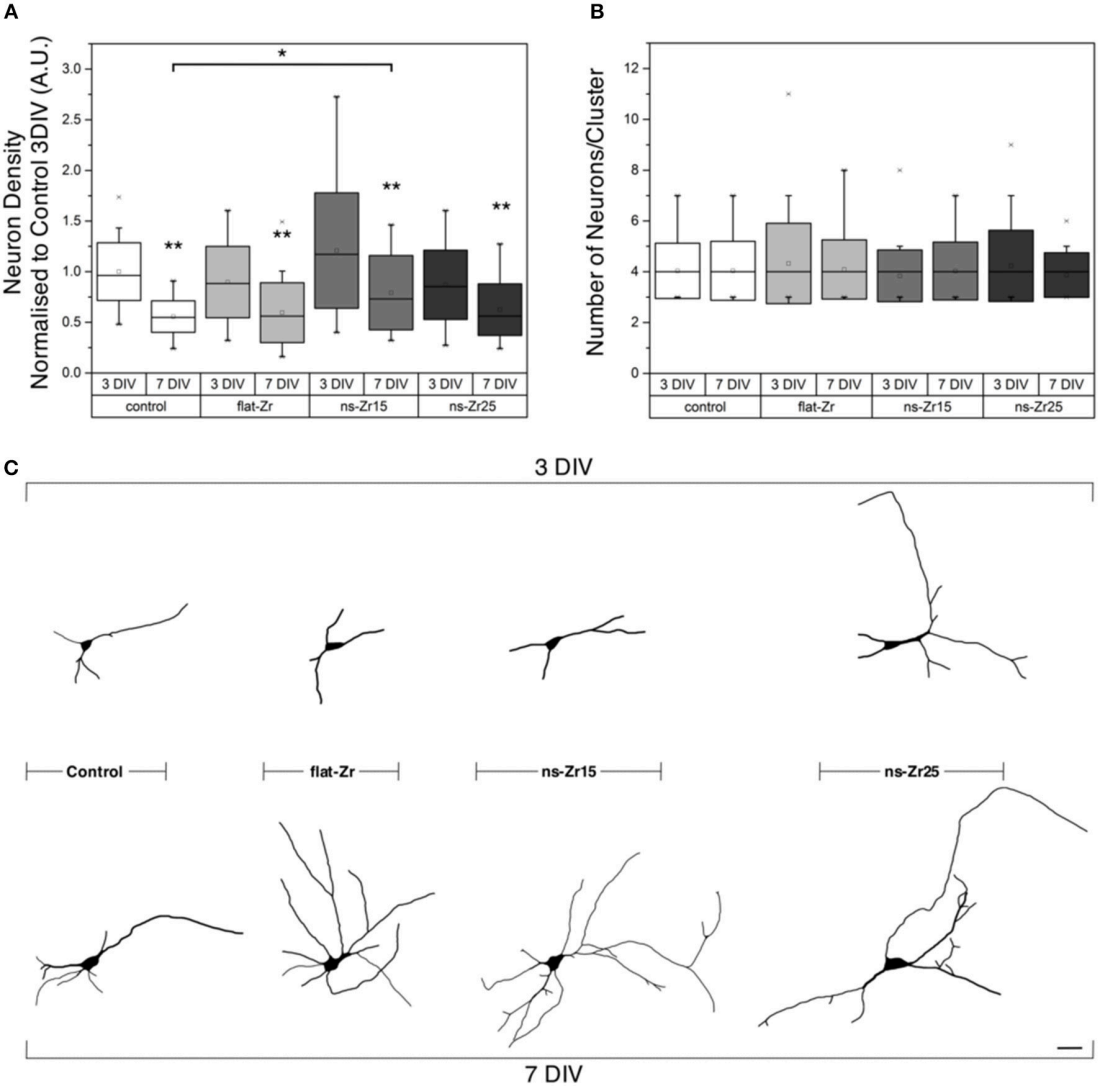
**Neuron density/viability, clustering, and neuronal morphology after interaction with the different surface topographies. (A,B)** Quantifications of the **(A)** number of neurons (normalized to Control 3 DIV) and **(B)** cell clustering of neuron populations grown on the different substrates were derived from NeuN staining and are represented in the boxplots and were derived from 3 individual experiments with total number of 355–651 analyzed cells (see methods for details). Images illustrating the appearance of typical neuron populations can be found in Figures 3A–H. **(C)** The graphics show representative examples of the neuronal morphology reconstruction (visualization obtained by lentiviral transduction with eGFP-VAMP2, details in the Methods). The scale bar represents 10 μm.

**Figure 3:**
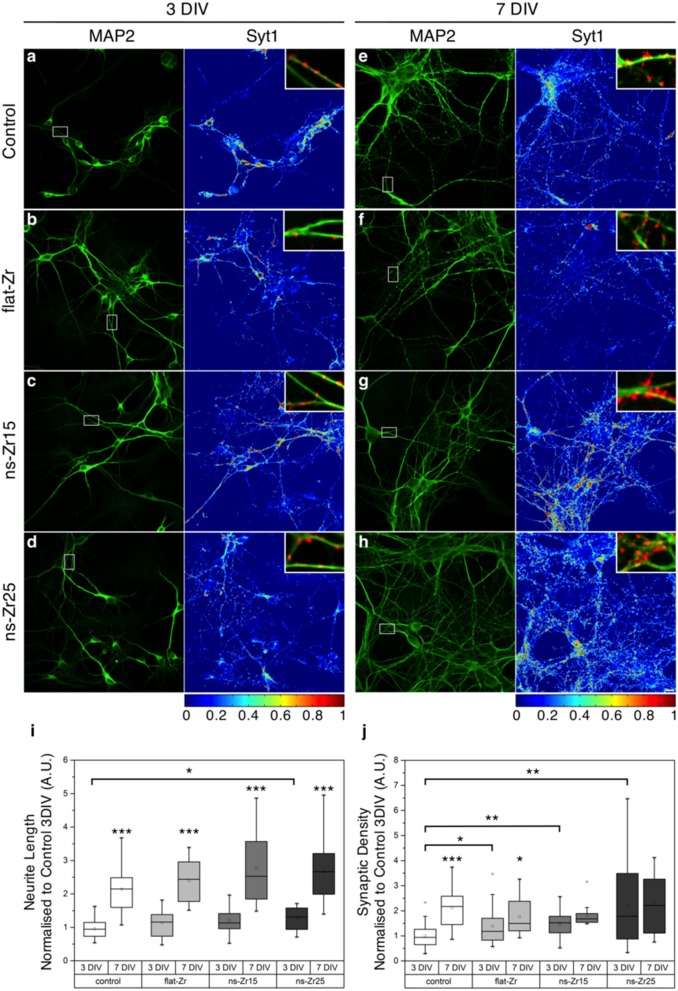
**Neurite outgrowth and synaptic density of the neural network in dependency of the substrate nanotopography. (A–H)** Confocal images of postnatal hippocampal cultures maintained *in vitro* for 3 **(A–D)** or 7 **(E–H)** days, fixed and stained with α-MAP2 (green, left panels) and α–synaptotagmin1 (Syt1)/p65 antibodies (right panels, color map as in LUT at the bottom). **(A–D)** At 3 DIV it can be noticed how in the Control (glass) and flat-Zr condition, the majority of Syt1 staining localizes inside cell bodies (exemplary zones marked in **(A–D)** with black asterisks), indicating an immature stage, in which synaptic proteins are still inside the endoplasmatic reticulum and Golgi apparatus to complete synthetisation. On ns-Zr15 and in particular on ns-Zr25, an increasing number of Syt1-positive puncta that can be considered as bona fide presynaptic boutons is visible. The somatic staining is less intense and present only in few somata with respect to Control or flat-Zr. **(E–H)** At 7 DIV a huge spread of dendritic trees (green) is clearly noticeable in all conditions if compared to 3 DIV and presynaptic bouton staining is elevated with respect to the 3 DIV. The vastest maturation paralleled by strongest development of presynaptic terminals can be noticed in ns-Zr25. The scale bar equals 10 μm. **(E–H)** The insets display details taken from the squared area indicated in the left panels to illustrate some representative presynaptic boutons (red: Syt1), juxtaposed to dendrites (green: MAP2) in the different conditions. **(I,J)** The plots (see methods for details) show the corresponding global statistics obtained from 3 individual experiments for **(I)** the neurite outgrowth (with a total of 10–20 analyzed fields) and **(J)** the synaptic density (with a total of 15–34 analyzed fields).

To evaluate the ability of these substrates to affect neuronal cell adhesion, viability, morphology (Figure 2), and neurite outgrowth (Figure 3), a fixed numbers of neonatal primary hippocampal cells (Postnatal day (P2), see methods for details) were plated onto cluster-assembled zirconia surfaces. Two different roughnesses (ns-Zr15, ns-Zr25) were used with flat surfaces (Control glass coverslips and flat-Zr) as control.

Initially the adhesion and viability of cells at day 3 and day 7 *in vitro* (3 DIV, respectively 7 DIV) were tested by looking at their density, spatial distribution and morphological appearance (Figures 2A–C, typical representations of the neuronal populations in the different conditions can be found in the panel of Figures 3A–H). The determination of the density of the neuronal population was carried out not only because it gives an estimate of cell adhesiveness and viability but also because this parameter affects neuronal maturation and network activity (Cullen et al., 2010; Biffi et al., 2013). Therefore, a careful control across different samples and experimental conditions was needed. No significant difference in this neuron density was found between the control and zirconia surfaces at 3 DIV with a small inter-sample variability (3 DIV Neuron Density normalized to Control 3 DIV ± *SD*: Control = 1 ± 0.28, flat-Zr = 0.90 ± 0.35, ns-Zr15 = 1.21 ± 0.57, ns-Zr25 = 0.87 ± 0.34; *n* = 355–651 cells from 3 independent experiments, all *p*-values > 0.05, Wilcoxon rank-sum test vs. Control). At 7 DIV the neuron density showed a general decrease with respect to the earlier time point for all conditions (Figure 2A). This is an expected finding which reflects the loss of a fraction of neuronal cells during *in vitro* culturing observed before (Oppenheim, 1991; Porter et al., 1997). The cell number on the ns-Zr15 after 7 DIV was significantly higher compared to the Control condition (7 DIV Neuron Density normalized to Control 3 DIV ± *SD* (percentage loss vs. 3 DIV): Control = 0.56 ± 0.16 (−44%), flat-Zr = 0.60 ± 0.30 (−33%), ns-Zr15 = 0.79 ± 0.37 (−35%), ns-Zr25 = 0.63 ± 0.25 (−28%); *n* = 355–651 cells from 3 independent experiments, ns-Zr15 vs. Control *p* = 0.03; *p* > 0.05 for all other substrates, Wilcoxon rank-sum test vs. Control). Regarding the spatial distribution and the appearance of cell clusters, our data did not indicate a significant difference between the conditions and/or time points (3 DIV Number of Neurons/Cluster ± *SD*: Control = 4.0 ± 1.1, flat-Zr = 4.3 ± 1.6, ns-Zr15 = 3.8 ± 1.0, ns-Zr25 = 4.2 ± 1.4; 7 DIV Number of Neurons/Cluster ± *SD*: Control = 4.0 ± 1.2, flat-Zr = 4.1 ± 1.2, ns-Zr15 = 4.0 ± 1.1, ns-Zr25 = 3.9 ± 0.9; *n* = 355–651 cells from 3 independent experiments, *p* > 0.05 vs. Control for conditions, Wilcoxon rank-sum test). This excludes a prominent effect of the different surface roughnesses on the migration of the hippocampal neurons and subsequent cell clustering (Figure 2B).

To get a first glance and impression of the neuronal morphology on the different substrates we transduced neurons with viral vectors expressing the fluorescent protein VAMP2-eGFP. The fluorescence of the transduced neurons rendered the identification of dendrites and axons easy (Sampo et al., 2003). The comparison of the substrates and time points suggested that, already at day 3 from plating, neurons displayed a more pronounced mature neuronal phenotype when grown on the ns-Zr25 surfaces with respect to the other experimental conditions. This differentiative behavior was clearly enhanced at 7 DIV (Figure 2C), resulting in a highly polarized phenotype with clearly distinguishable axons and axonal presynaptic varicosities which is characteristic for mature neurons. In the other conditions (flat surfaces and ns-Zr15), consistent with previous results in standard culture substrate condition (Bose et al., 2000), the neurons still retained a more immature morphology.

A quantification of the neurite outgrowth (by a staining against the neurite and dendrite marker MAP2, representative examples are shown in the panel of Figures 3A–H) confirmed furthermore that the neurons grown on ns-Zr25 expanded their neurites already stronger at 3 DIV, compared to the Control condition (3 DIV Neurite Length Normalized to Control 3 DIV ± *SD*: Control = 1 ± 0.28, flat-Zr = 1.18 ± 0.41, ns-Zr15 = 1.26 ± 0.45, ns-Zr25 = 1.30 ± 0.36, ns-Zr25 vs. Control *p* = 0.036; *p* > 0.05 for all other substrates, *n* = 10–20 fields from 3 independent experiments, Wilcoxon rank-sum test). In all conditions an expected branching of the neuritic/dendritic tree was observed toward 7 DIV (7 Div > 3 DIV, *p* < 0.01 for all substrates, Wilcoxon rank-sum test vs. same substrate at 3 DIV) but it remained most pronounced on the nanostructured substrates (7 DIV Neurite Length Normalized to Control 3 DIV ± *SD*: Control = 2.23 ± 0.74, flat-Zr = 2.49 ± 0.71, ns-Zr15 = 2.87 ± 1.12, ns-Zr25 = 2.79 ± 0.93; *p* > 0.05 for all substrates, *n* = 10–20 fields from 3 independent experiments, Wilcoxon rank-sum test) (Figure 3I).

Altogether, these results suggest that in particular the ns-Zr25 surface can accelerate neuronal cell development and maturation.

### The nanostructured zirconia substrate accelerates synaptogenesis

These interesting observations prompted us to test whether the interaction of the neurons with the nanotopography affects the synaptogenesis. The functionality of neuronal cells depends on a complex synaptic protein machinery which regulates e.g., vesicle trafficking. In developing neurons this machinery appears before the synapses are even operative and electrically active (Greif et al., 2013). Therefore, as a read-out we counted synapses present in the different culturing conditions. Presynaptic varicosities were immunolabeled with an antibody against synaptotagmin-I/p65 and juxtaposed to dendrites (MAP2) (Figures 3A–H). Synaptotagmin-I/p65 is presynaptic marker and an integral synaptic vesicle protein (Matthew et al., 1981; Greif et al., 2013) involved in determining neuronal polarity and axon formation/specification (Greif et al., 2013; Inoue et al., 2015).

Compared to the Control condition, the synaptic density for neurons grown on ns-Zr15 and ns-Zr25 was already highly significantly increased at 3 DIV, with the highest value and level of significance observed for the latter one (3 DIV Synaptic Density Normalized to 3 DIV Control ± *SD*: Control = 1 ± 0.45; flat-Zr = 1.38 ± 0.76, *p* = 0.05; ns-Zr15 = 1.45 ± 0.59, *p* = 0.01; ns-Zr25 = 2.21 ± 1.60, *p* = 0.002, *n* = 15–34 fields from 3 independent experiments, Wilcoxon rank-sum test vs. Control 3 DIV) (Figure 3J). The synaptic density remained on their high levels on ns-Zr15 and ns-Zr25 with only minor further, not significant, increases suggesting a maturation of the synaptic connections. Coming from the lower 3 DIV level, the synaptic density augmented also on the flat surfaces over time toward 7 DIV, as to be expected (Bose et al., 2000) (7 DIV Synaptic Density Normalized to 3 DIV Control ± *SD*: Control = 2.11 ± 0.77; flat-Zr = 1.77 ± 0.73, *p* = 0.1; ns-Zr15 = 1.80 ± 0.43, *p* = 0.25; ns-Zr25 = 2.32 ± 1.10, *p* = 0.52, Wilcoxon rank-sum test vs. Control 7 DIV; 7 DIV > 3 DIV, Control *p* = 8.3 ^*^ 10^−8^, flat-Zr *p* = 0.02, ns-Zr15 *p* = 0.12, ns-Zr25 *p* = 0.46, *n* = 15–34 fields from 3 independent experiments, Wilcoxon rank-sum test vs. same substrate at 3 DIV) (Figure 3J).

The data indicate an acceleration of the synaptogenic processes in the neurons interacting with the nanotopographic features.

### Vast impact of the neuron/nanotopography interaction on the neuronal protein profile

After these results demonstrating the capacity of nanostructured zirconia surfaces to promote synaptogenesis, we wanted to understand the maturation-promoting effect of the nanostructured zirconia topography on the cellular program in a more general way. For this purpose we were benefitting from the potential of the SCBD nanofabrication technique to provide large macroscopic areas with a defined nanostructure. This allowed a profound confrontation of the protein profile of neurons interacting for 3 days with the ns-Zr25, i.e., the substrate found to produce the largest enhancement in neurito-/synaptogenesis (Figures 3I,J), with those in the standard control culture condition (Control, glass coverslips) and flat-Zr, via quantitative shotgun proteomic analysis.

**Figure 4:**
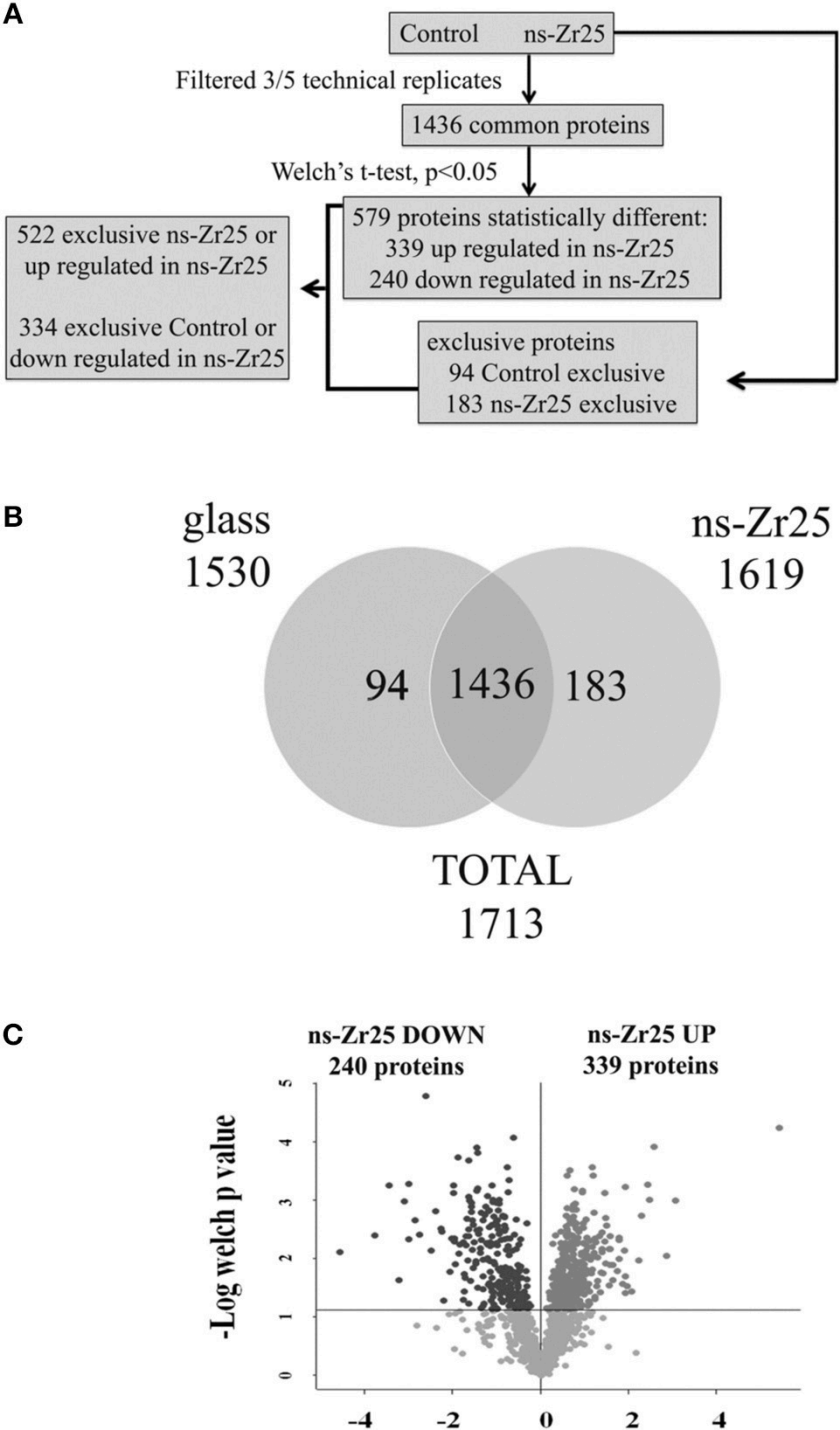
**Proteomic workflow and analysis for the comparison between ns-Zr25 and Control. (A)** Work flow of the proteomic approach. A shotgun proteomic analysis was performed on the hippocampal neurons cultured for 3 days either in the Control condition or on the nanostructured zirconia surface with a roughness R_q_ of 25 nm rms. Statistical analyses were performed using the Perseus software (version 1.4.0.6, www.biochem.mpg.de/mann/tools/). **(B)** Venn diagram of the comparison between cells grown on ns-Zr25 and in the Control condition. Only proteins present and quantified in at least 3 out of 5 technical repeats were considered as positively identified in a sample and used for statistical analyses. **(C)** Vulcano plot of the proteins differentially expressed. Proteins were considered differentially expressed if they were present only in ns-Zr25 or Control or showed significant *t*-test difference (cut-off at 5% permutation-based False Discovery Rate). The data points that are above the *p*-value line (*t*-test value cut off = 0.0167) in the volcano plot represent the proteins that were found to be differentially expressed in these two conditions upon treatment with large magnitude fold changes and high statistical significance: In dark gray the proteins downregulated, in light gray the upregulated.

The work flow of the proteomic approach for the comparison between ns-Zr25 and Control is reported in Figure 4A. Only proteins present and quantified in at least 3 out of 5 technical repeats were considered as positively identified and used for statistical analyses (Figures 4A,B). Proteins were considered differentially expressed if they were present only in ns-Zr25 or Control or showed significant *t*-test difference (cut-off at 5% permutation-based False Discovery Rate) (Figure 4C, Volcano plot). 522 proteins were upregulated or present only in cells grown on ns-Zr25, while 334 proteins were downregulated in cells grown on ns-Zr25 or were present only in cells grown in the Control condition (Tables S1, S2).

**Figure 5:**
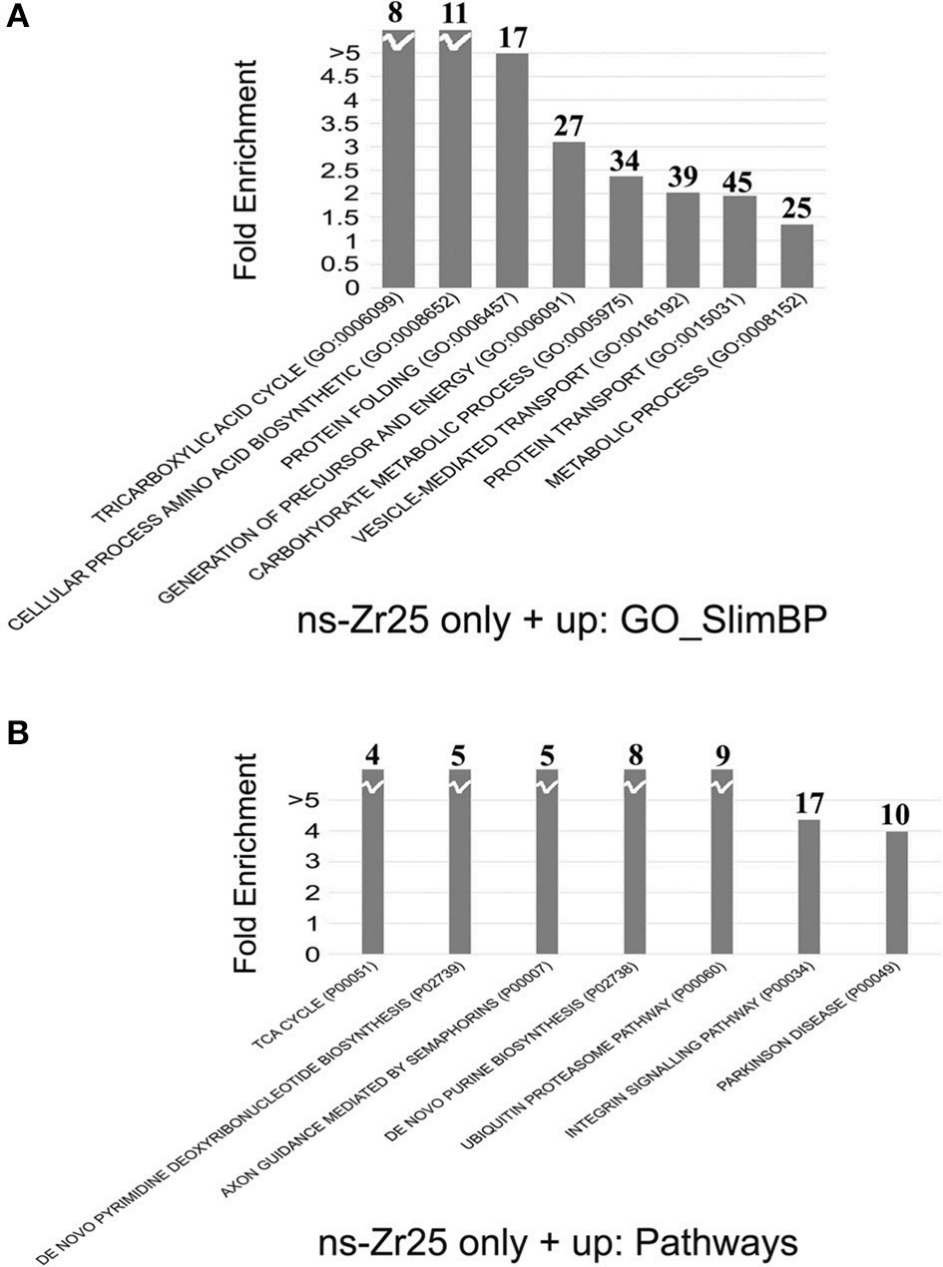
**Gene annotation enrichment analysis for the comparison between ns-Zr25 and Control. (A,B)** The analysis was carried out on proteins upregulated or expressed only in ns-Zr25. The proteins differently expressed were clustered according to their functions using the Panther platform (Version 10.0 release date April 25, 2015) filtered for significant Gene Ontology terms: **(A)** Biological Process (GO-SlimBP) and **(B)** pathways using a *p* value < 0.05. The fold enrichment value is reported in the y-axis. The numbers in bold above each bar indicates the number of genes enriched in the analysis.

**Figure 6:**
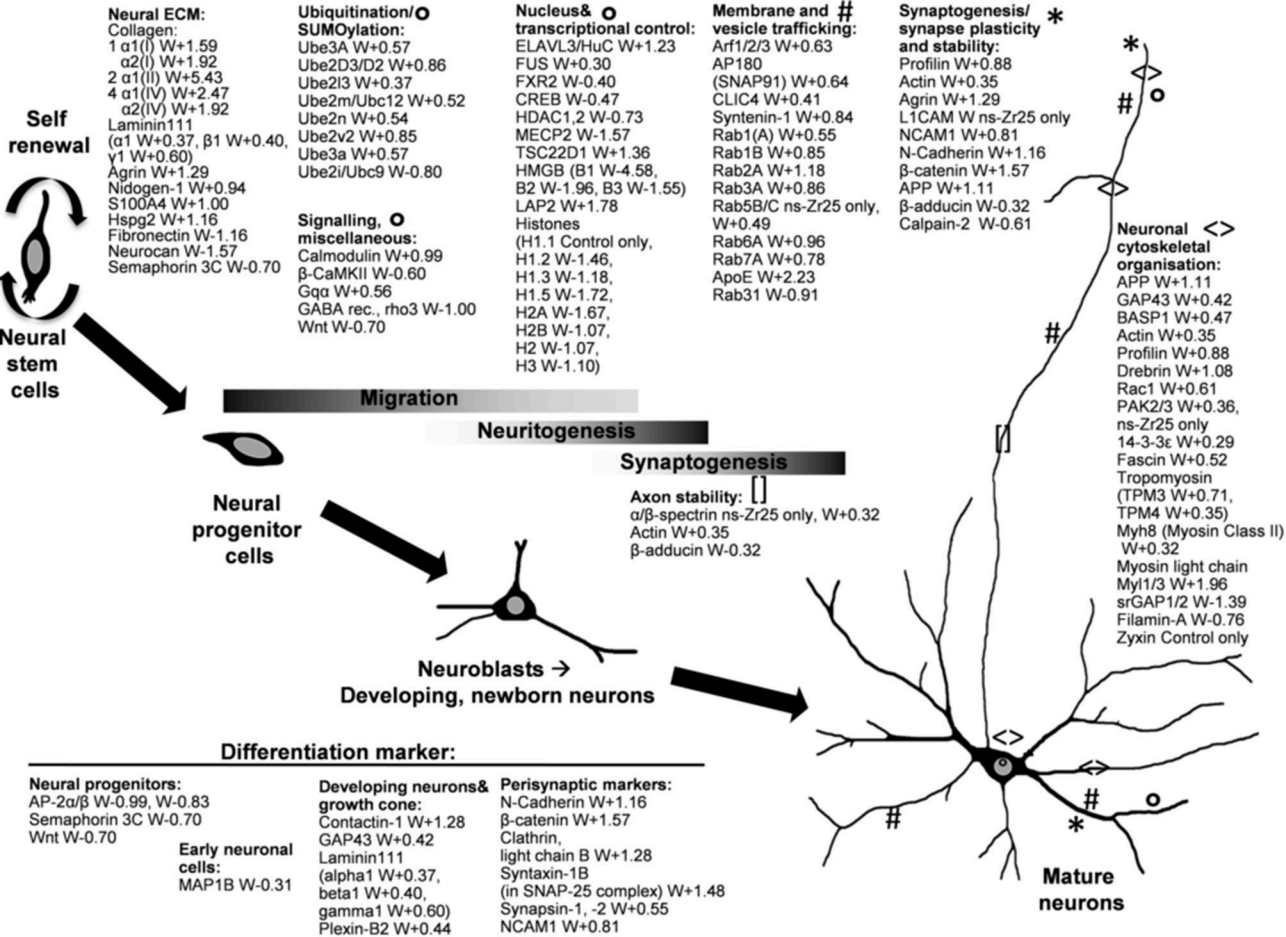
**Selection of changes in the cellular program induced by the interaction with the nanostructured zirconia surface (ns-Zr25) compared to the Control**. The graphical illustration accentuates various exemplary proteins which were altered in their expression levels in neurons grown on ns-Zr25 compared to the Control condition and are known to have prominent roles in processes important for neurogenic development and or integrin adhesome-, cytoskeleton- mechanotransduction-related processes. Further information on many of these proteins is provided in the main text. The numbers behind the protein names indicates the Welch difference (W). Complete lists of the differentially expressed proteins can be found in Tables S1, S2, with IAC proteins [according to Winograd-Katz et al. (2014)] marked in bold. Further IAC proteins [according to Geiger and Zaidel-Bar (2012)] are listed in Table S5.

Gene annotation enrichment analysis was carried out by Panther software to cluster enriched annotation groups within the set of differentially expressed proteins in terms of the highest enrichment score (Figure 5). Among these categories, several of them reflect an increase of mitochondrial activity. More than 31% of the proteins induced by ns-Zr25 (163 out of 522, marked in gray in Tables S1, S2) are mitochondrial proteins mainly involved in the generation of precursor metabolites and energy, suggesting an increase in mitochondrial activity (Figure 5A). This is intriguing because neuronal activity and especially synaptic transmission requires a considerable energy supply. For a sufficient provision of energy, mitochondria and their translocation to synaptic boutons are indispensable. An impaired energy supply to synapses can cause neuronal pathologies (Harris et al., 2012; Sheng and Cai, 2012; Sheng, 2014). Furthermore, confirming the data regarding synaptic density, important proteins for synaptic transmission and vesciculation are abundantly enriched (Figure 5A). In line with the nature of the biophysical nanotopographical signal input, the proteomic data of the neurons grown on ns-Zr25 also propose a strong involvement of axon guidance and integrin signaling-related processes (Figure 5B) known to depend predominantly on the features of the neuronal microenvironment. We will further specify the latter two aspects in the alterations of the cellular program of neurons interacting with ns-Zr25 in the discussion (examples are summarized thematically in Figure 6).

To account for changes being due to the nanotopography alone and not due to the zirconia material itself, a similar proteomic analysis was carried out comparing ns-Zr25 and flat-Zr (Figure S1). 347 proteins were upregulated or present only in cells grown on ns-Zr25, while 637 proteins were downregulated in cells grown on ns-Zr25 or were present only in cells grown on flat-Zr (Figure S1A and Tables S3, S4). Interestingly enough, the Gene annotation enrichment analysis shows a significant increase of differentially expressed proteins involved in cell-matrix adhesion (Figure S1B) and the integrin signaling pathway (Figure S1C) for the neurons that interact with ns-Zr25 instead of the flat-Zr.

This strongly suggests that maturation-promoting mechanotransductive events might be triggered specifically by the nanotopography and not by the material.

### The neuron/nanotopography interaction promotes the generation of functional neural networks

**Figure 7:**
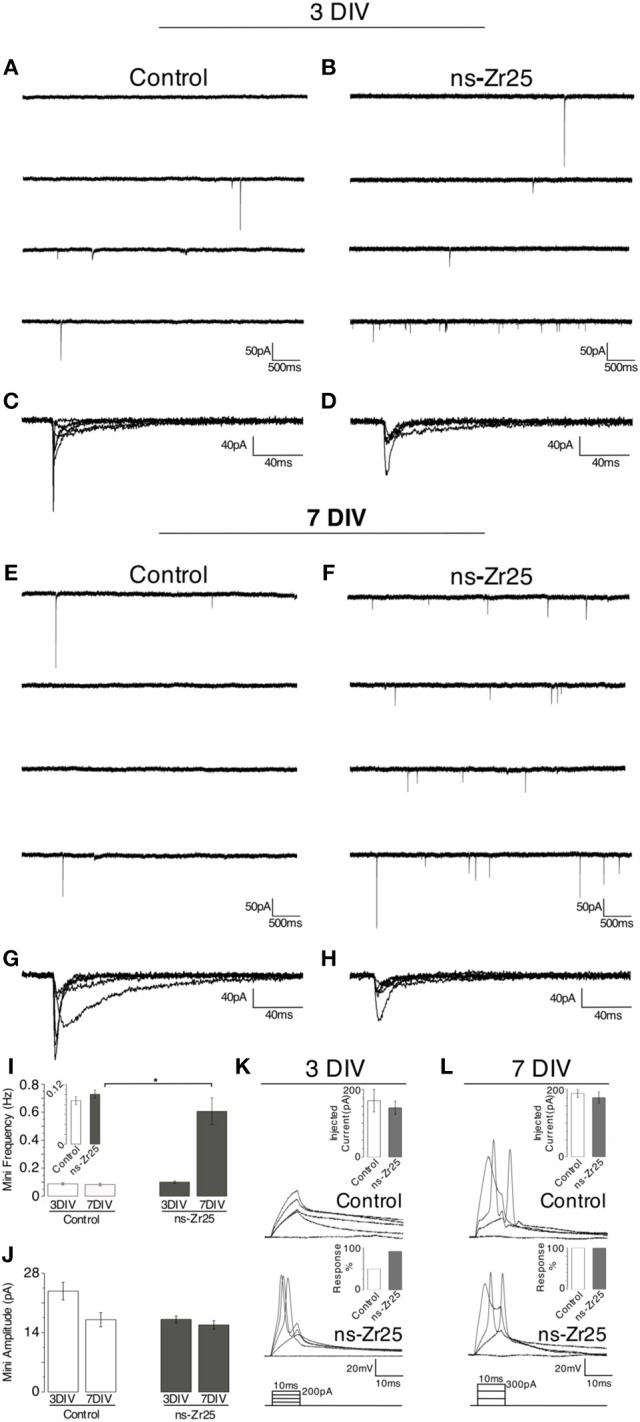
**Electrophysiological recordings from cultured hippocampal neurons on flat glass (Control) or the nanostructured zirconia surface (ns-Zr25)**. Hippocampal neurons were plated on glass (Control) or nanostructured zirconia surfaces (ns-Zr25). Electrophysiological recordings (see methods for details) were done after 3 **(A–D,K)** or 7 **(E–H,L)** days of *in vitro* maturation on these surfaces. Exemplary miniature current traces recorded from neurons plated on Control or ns-Zr25 surfaces after 3 DIV are shown in panels **(A)** and **(B)**, and after 7 DIV in panels **(E)** and **(F)**. Bars in graph **(I)** represent the corresponding mean frequency of miniature postsynaptic currents (mPSCs). At 3 DIV no significant difference between Control (white bars) and ns-Zr25 (gray bars) was found, even if a trend (see also the inset) of an increased frequency in the ns-Zr25 condition starts to emerge (Control = 0.087 ± 0.008, *n* = 8 cells; ns-Zr25 = 0.101 ± 0.008, *n* = 14 cells; *p* > 0.05; Wilcoxon rank-sum test, error bars are sem). This tendency stands out at 7 DIV, at this stage a significant increase in mPSCs frequency in neurons grown on ns-Zr25 surfaces was found (Control = 0.082 ± 0.009, *n* = 8 cells; ns-Zr25 = 0.606 ± 0.094, *n* = 10 cells; *p* < 0.05; Wilcoxon rank-sum test, error bars are SEM). Representative events from exemplary neurons are overlapped in panels **(C,D,G,H)** to show the variability in shape and amplitude in the different conditions. **(J)** The bar panel displays the mean of the amplitude mPSCs in the different conditions. The obtained data show a trend in the Control condition; with a higher mean amplitude of the miniatures in immature neurons (3 DIV) and a decrease over maturation (7 DIV) as expected from previous reports (Bose et al., 2000). On ns-Zr25 stable mean amplitude was observed over maturation with a value in the range of the mean value seen for the mature neurons grown in the Control condition at 7 DIV (3 DIV mean amplitude: Control = 23.76 ± 2.09; ns-Zr25 = 17.11 ± 0.8; 7 DIV mean amplitude: Control = 17.04 ± 1.68; ns-Zr25 = 15.8 ± 0.1). Panels **(K)** and **(L)** represent exemplary membrane voltage recordings from individual neurons cultured in different conditions. The insets display the injected current thresholds and the percentage of responding cells. When triggered to fire action potentials, by current injections steps (injected current protocol scheme on the bottom), **(K)** young neurons (3 DIV) cultured on ns-Zr25 surfaces demonstrate an enhanced excitability compared to neurons maintained in Control condition. **(L)** At 7 DIV Control neurons acquired excitability comparable to the ns-Zr25 condition (injected current protocol scheme on the bottom).

The data on neurite outgrowth, synaptic density and the numerous hits from the proteomic analysis strongly indicate a promotive effect of the neuron/nanotopography interaction on the build-up of a functional neural network. To further validate if this accelerated and enhanced appearance of neurites/dendrites and presynaptic boutons on the nanostructured surfaces and the extensive alterations in the neuronal proteome indeed led to active and functional synaptic units, we evaluated the neuronal and synaptic activity by electrophysiological experiments (whole-cell patch clamp recordings, Figure 7).

Cultured hippocampal neurons are known to become excitable and to generate action potentials beginning from 3 DIV (Cohen et al., 2008). This activity, highlighted by the presence of individual or small sequences of spontaneous action potentials, arises from the input of the developing synaptic connectivity, a behavior which is enhanced during *in vitro* maturation (Ichikawa et al., 1993; Craig et al., 2006).

To analyse the timing and extent of the synaptic network maturation, we searched for the presence of spontaneous miniature postsynaptic currents (mPSCs, minis; voltage-clamp recordings; tetrodotoxin, TTX, 1 μM). These recordings were run on the hippocampal neurons after 3 DIV and 7 DIV on ns-Zr25, using the canonical culture condition (Control) as reference. In both plating conditions, some low frequency miniatures events could be detected beginning at day 3 in culture (3 DIV, average mini frequency ± SEM: Control = 0.088 ± 0.008 Hz, *n* = 8; ns-Zr25 = 0.101 ± 0.008 Hz, *n* = 14; ns-Zr25 vs. Control, *p* = 0.76, Wilcoxon rank-sum test) (Figures 7A–D,I). When recordings were performed at day 7, a significant difference (> 7-fold) between the two growing conditions was found (7 DIV, average mini frequency ± SEM: Control = 0.083 ± 0.009 Hz, *n* = 8; ns-Zr25 = 0.607 ± 0.095 Hz, *n* = 10; ns-Zr25 vs. Control, *p* = 0.04, Wilcoxon rank-sum test) (Figures 7e–H,I), with a clear and significant increase (~6-fold) in mini frequency detected only for neurons grown on the ns-Zr25, while no change was recognizable in the Control condition (ns-Zr25 7 DIV vs. 3 DIV, *p* = 0.02; Control 7 DIV vs. 3 DIV, *p* = 0.80; Wilcoxon rank-sum test).

To evaluate the quantal postsynaptic responsiveness we analyzed the amplitude of miniature currents in all conditions (Figures 7C,D,G,H,J). The mini amplitude was comparable to what was found in previous experiments (Bose et al., 2000) and there were no significant differences between the conditions (average mini amplitude ± SEM: Control 3 DIV, 23.8 pA ± 2.1; Control 7 DIV, 17.0 pA ± 1.7; *n* = 8 recordings each; average mini amplitude ns-Zr25 3 DIV, 17.1 pA ± 0.8; ns-Zr25 7 DIV 15.8 pA ± 1.0; *n* = 14, respectively *n* = 10 recordings; *p* > 0.05 for all conditions vs. Control Wilcoxon rank-sum test). However, it is interesting to note that there was a slight, even though not significant, decrease along *in vitro* maturation in the Control condition, whereas for the neurons on ns-Zr25 the amplitudes remained stable on a lower level.

Since this anatomical and functional developmental profile of neurons should be matched by a change in excitability, we tested the ability of neurons to fire action potential in the same experimental conditions. Therefore, the neurons were stimulated by a series of incremental current injections (1–300 pA; current-clamp experiments) and the likelihood of action potential firing was recorded. At day 3, a larger proportion of cultured hippocampal neurons grown on ns-Zr25 were capable of responding to current pulses with *bona fide* action potentials than in control conditions (3 DIV, % of responding neurons (current threshold ± SEM): Control = 50% (166.7 pA ± 33.3), ns-Zr25 = 92% (145.5 pA ± 19.6); Control *n* = 8 recordings, ns-Zr25 *n* = 12 recordings) (Figure 7K). As expected from previous reports (Cohen et al., 2008), when neurons were tested at day 7, even on glass coverslips all neuron responded by generating action potentials [7 DIV; 100% of responding neurons in Control (187.5 pA ± 12.5) and ns-Zr25 (175.0 pA ± 16.4); *n* = 8 recordings each], but neurons grown on ns-Zr25 still displayed a lower current threshold for firing (Figure 7L). This suggests that the developmental profile for voltage activated ion channels was still enriched by the interaction with the nanostructured zirconia substrate.

Altogether, these electrophysiological results show that neurons grown on ns-Zr25 are not only viable, but also their maturation profile is significantly enhanced, with a more profound morphological and functional synaptic integration. The overall behavior of the neurons interacting with the ns-Zr25 surface is highly compatible with the proteomic profile and a more mature condition of the neural network.

## Discussion

In recent years a considerable amount of effort has been devoted to the development of nanoengineered surfaces which resemble ECM topographical features and determine cell fate by modulating cellular differentiation processes (Kim et al., 2012; Gasiorowski et al., 2013; Mendes, 2013; Dalby et al., 2014; Murphy et al., 2014; Chen et al., 2015). Clearly these artificial substrates have an important potential in the framework of regenerative medicine. Regarding the molecular mechanism, the potential of these biomaterials arises from their ability to modify cell adhesion- and mechanotransduction-dependent actions (Dalby et al., 2014; Murphy et al., 2014; Chen et al., 2015) but specific details remain elusive.

The nanotechnological approach exploited by our group is based on the production of such nanoengineered surfaces with the help of supersonic cluster beam deposition of zirconia nanoparticles (Wegner et al., 2006). The SCBD technique allows to create nanostructured films with controllable and reproducible nanotopographical features (Figure 1) (Wegner et al., 2006; Podestà et al., 2015) equipped with characteristics and dimensions that mimic those found at the nanoscale level in the ECM (Gasiorowski et al., 2013). We have recently shown that these surfaces produced by SCBD have the capacity to modulate crucial cell adhesion-related parameters, in particular the IAC nanoarchitecture/dynamics and composition and consequentially the cellular mechanobiology. Moreover, it emerged that these mechanotransductive processes promote neuronal differentiation in the neuron-like PC12 cells (Schulte et al., 2016).

In the present work, we have analyzed whether nanostructured zirconia surfaces can foster differentiation processes in a clinically relevant primary neuronal cell model, i.e., neuronal cells obtained from the new-born rat hippocampus (postnatal day 2). At this stage these neurons are still immature and once dissociated they completely lose their anatomical and functional characteristics to start a “new life” *in vitro*. Numerous reports have shown that cultured primary hippocampal neurons develop a polarized shape with dendrites and an axon, express voltage-activated ion channels and become excitable. The coupling of functional synaptic contacts follows these initial maturative steps resulting in the formation of well-integrated neural networks (Raineteau et al., 2004; Cheyne et al., 2011).

*In vivo*, the formation of these networks, especially the axon guidance and synaptic plasticity, depends on extracellular cues that lead to complex changes of the cellular program realizing the neuronal maturation (Benson et al., 2001; Pizzorusso et al., 2002; Graf et al., 2004; Nam and Chen, 2005; Sara et al., 2005; Craig et al., 2006; Dityatev et al., 2010; Myers et al., 2011; Vitriol and Zheng, 2012). On standard plastic petri dishes and glass coverslips with unnaturally flat and featureless surfaces some of these events can be rather slow and the formation of a mature synaptic network usually requires 1–2 weeks (Chiappalone et al., 2006; Wagenaar et al., 2006). As illustrated in Figure 2C, we found that in particular on substrates with the roughness R_q_ of 25 nm rms neurons exhibited a mature phenotype with an increase in neurite outgrowth and synaptic varicosities already after 3 DIV (Figures 3I,J). At this stage, on ns-Zr25 a large fraction of neurons was also found to be already excitable. As expected from previous studies (Bose et al., 2000), this functional behavior was not found in control cultures grown on glass coverslips at this early stage (Figure 7K). Furthermore, on ns-Zr25 the presence of spontaneous synaptic currents (minis), indicative of fully formed and active synaptic contacts, showed an incremental over time, reaching a consistent difference over control cultures after 7 DIV (Figure 7I).

The strong impact of the neuron/ns-Zr25 interaction on the neuronal morphological and molecular phenotype indicates that the acceleration of the maturative steps emanates from a direct or indirect activation of specific genetic programs. We were able to collect sufficient cellular material to profoundly analyse the impact of the neuron/ns-Zr25 interaction on the cellular program via label-free shotgun proteomics due to the advantage of the SCBD nanofabrication technique to allow the production of large macroscopic areas with a defined nanostructure.

These means enabled us to unveil the large influence of this interaction on the neuronal proteome (e.g., >850 differentially expressed proteins on ns-Zr25 vs. Control, see Figure 4) showing alterations broadly congruent with the demonstrated accelerated induction of neurito-/synaptogenesis and neuronal network maturation. Moreover, the data suggest a strong impact of the neuron/nanotopography interaction on cell adhesion processes and in particular on axon guidance and integrin signaling pathways (Figures 4–6, Figure S1, Tables S1–S5) whose regulation *in vivo* is predominantly substrate-dependent (Benson et al., 2001; Craig et al., 2006; Dityatev et al., 2010; Myers et al., 2011; Vitriol and Zheng, 2012).

To illustrate the effect of the nanotopography on the hippocampal neurons we highlight various examples of proteins, focusing on the comparison between ns-Zr25 and Control (summarized thematically in Figure 6, the complete lists can be found in Tables S1, S2). The indicated proteins are known to have essential roles in versatile cellular processes that strongly influence neuronal functioning, neurogenesis, synaptogenesis and neuronal maturation, and/or have significance regarding IAC- and mechanobiology-related aspects.

First of all, the proteomic profile validated extensively the general shift toward neuronal cells that are in a further advanced stage of neurogenic development and neuronal maturation triggered by the nanostructured surface. Markers for neural progenitors (e.g., AP-2 and semaphorin 3C) or early neuronal cells (MAP1B) are strongly downregulated. The elevated status of synaptogenesis and maturation is instead confirmed by the upregulation of many prominent markers for developing neurons (contactin-1, laminin 111), growth cones (GAP43, BASP1), neurite/axon outgrowth, synapses and mature neurons (e.g., NCAM, N-Cadherin, β-catenin, Clathrin light chain B, syntaxin-1B, synapsin-1).

Neurito/dendrito/axonogenesis and subsequently the formation of synapses are crucial events during the development of neurons. Many proteins with well-documented tasks in the regulation or realization of these processes are upregulated in the hippocampal neurons on ns-Zr25. APP, to start with, represents a key regulator in neural development which therein orchestrates versatile signaling cascades and biological functions (Nicolas and Hassan, 2014). Neurite/axon outgrowth/guidance, growth cone advancement (Dent et al., 2011) and synaptogenesis (Nelson et al., 2013) require a highly coordinated spatiotemporal regulation of cell adhesion and the cytoskeletal dynamics (Fletcher and Mullins, 2010; Dent et al., 2011). Consistently, various proteins essentially involved in the regulation of the neuronal cytoskeletal organization are upregulated (e.g., profilin, drebrin, Rac1, PAK2/3, fascin, 14-3-3ε, β-spectrin, tropomyosins). Profilin (Birbach, 2008), drebrin (Sekino et al., 2007), Rac1 (Aoki et al., 2004; Schwamborn and Püschel, 2004), and PAKs (Kreis and Barnier, 2009) have a strong impact on neuronal morphology and the plasticity of dendritic spines and synapses. Fascin contributes to neuritogenesis by its actin-bundling function in growth cone filopodia (Cohan et al., 2001; Dent et al., 2007, 2011) and is critical for the regulation of FA and stress fiber dynamics. Its depletion decreases the FA turnover (Elkhatib et al., 2014). 14-3-3ε controls NCAM/spectrin-dependent axon outgrowth (Ramser et al., 2010) and presynaptic functions (Broadie et al., 1997) and neurogenesis (Toyo-oka et al., 2014), through actin cytoskeleton-mediated processes. Spectrin again is an actin-binding protein and important for the axon (Xu et al., 2013) and synapse stability and function (Pielage et al., 2005) supporting the formation of highly ordered cytoskeletal structures within the axon shaft. Also the downregulated β-adducin plays a complex not yet fully understood role in synapse dynamics, more precisely in the switch between synapse growth and elimination (Bednarek and Caroni, 2011; Pielage et al., 2011; Stevens and Littleton, 2011; Xu et al., 2013). Further downregulated proteins in this cytoskeletal context are WAVE and srGAP. In general, WAVE and the upregulated tropomyosins control in a reciprocal crosstalk the actin filament branching (Bugyi et al., 2010; Krause and Gautreau, 2014); for the latter one distinct roles in neurons for the different isoforms have been described (Schevzov et al., 2012). Interestingly, tropomyosin has been found to regulate mechanotransductive processes via sarcomer-like structures (Wolfenson et al., 2016). srGAPs are essential for the fine-tuning of the neurite leading process branching, modulating neuronal morphogenesis and migration (Pertz et al., 2008; Guerrier et al., 2009).

The cytoskeletal organization depends furthermore strongly on IAC composition/signaling which therefore plays a fundamental role in neuronal development (Robles and Gomez, 2006; Gupton and Gertler, 2010; Eva and Fawcett, 2014; Kerstein et al., 2015). A potential contribution of IAC- and mechanotransduction-related actions to the observed nanotopography-induced events becomes quite evident from the proteomic data. From the list of 63 proteins found consistently in 3 independent adhesome proteomic studies compared by Geiger and Zaidel-Bar (2012), 37 show a significant change in the expression level in the neurons interacting with the ns-Zr25 (Table S5). Moreover, 16 proteins indicated as adhesome components in a list published by Winograd-Katz et al. (2014) are altered in their expression (marked in bold in the Gene names column in Tables S1, S2). Among them is e.g., the downregulated zyxin, a LIM domain-containing IAC core protein (Horton et al., 2015) essential for actin bundle formation during focal adhesion (FA) maturation (Yoshigi et al., 2005). A strong modulation of the cell-matrix adhesion process (Figure S1B) and the integrin signaling pathway (Figure S1C) emerges from the proteomic comparison of neurons on ns-Zr25 and flat-Zr which further highlights the specific importance of the topography (with respect to the material itself) concerning the mechanotransduction aspect. Among the differentially expressed proteins 45 proteins of the Geiger and Zaidel-Bar list (Geiger and Zaidel-Bar, 2012) are represented (Table S5), e.g., various downregulated LIM domain-containing proteins whose recruitment to IAC during FA maturation is dependent on mechanical tension and actomyosin-mediated contraction (Schiller et al., 2011). From the 37 proteins found in the comparison ns-Zr25 vs. Control, 17 are differentially expressed in the same manner also in ns-Zr25 vs. flat-Zr (only 4 in an opposite manner). 36 proteins (marked in bold in the Gene names column in Tables S3, S4) from the Winograd-Katz et al. adhesome list (Winograd-Katz et al., 2014) can be found and, compared to ns-Zr25 vs. Control, 9 out of 16 proteins were differentially expressed in the same manner (only 3 in an opposite manner). Altogether, these results are in line with our findings in PC12 cells (Schulte et al., 2016) but further experiments need to address this aspect of mechanotransduction more profoundly also in these primary neurons.

The eventual destiny of neurons is to establish connections and communication with other neurons by the formation of functional synapses and the build-up of neural circuits. The synaptic density data and the electrophysiology showed that after 3 days on the maturation-promoting nanostructured zirconia the course is already largely set toward this. Later on after 7 DIV the neural network activity is indeed very high compared to the control condition. The same conclusion can also be deduced from the proteomic data. NCAM, L1CAM, N-Cadherin and β-catenin are known to play crucial roles in synaptogenesis and synapse function/plasticity, in particular also in hippocampal cells (Lüthl et al., 1994; Okuda et al., 2007; Arikkath and Reichardt, 2008; Giagtzoglou et al., 2009; Mendez et al., 2010) and are all strongly upregulated. Furthermore, the ratio of α- to β-CaMKII has been linked to the level of network activity (Thiagarajan et al., 2002). A high level of β-CaMKII indicates low network activity and congruently β-CaMKII is less present in the ns-Zr25 condition. Calmodulin itself is upregulated, in line with its essential function in calcium signaling-regulated synaptic plasticity (Wayman et al., 2008). In addition, Gαq is upregulated, a heterotrimeric G protein which regulates synaptic signaling by mediating the downstream effects of many neurotransmitters and hormones (Gerber et al., 2016). Also two members of the calpain family are downregulated (Capn2, calpain-2; Capns1, calpain 4). These proteases have versatile substrates that often have roles in the IAC, the actin cytoskeleton organization and/or in synaptic functioning. In particular the downregulation of calpain-2 is congruent with the observed results, as it is known to be a kind of molecular brake for synaptic plasticity and long-term potentiation (Baudry and Bi, 2016).

Also several important components of the axon and synapse microenvironment (Barros et al., 2011) are found to be upregulated, e.g., agrin (Bose et al., 2000; Karasewski and Ferreira, 2003; Martin et al., 2005; McCroskery et al., 2006), laminin-111 (Marangi et al., 2002; Turney and Bridgman, 2005) and some collagens. In particular, collagen IV plays an important role in axon outgrowth and synaptic maturation (Fox et al., 2007; Barros et al., 2011). Another basement membrane protein found to be strongly expressed is Nidogen-1, a prominent regulator of synaptic plasticity and excitability in hippocampal neurons (Vasudevan et al., 2010). S100A4 and HSPG(Lutolf et al., 2009) are upregulated which, in a cooperative manner, are potent inducer of neurite/axon outgrowth in hippocampal neurons (Novitskaya et al., 2000; Kiryushko et al., 2006). In this context, it is in line that neurocan instead is downregulated in the ns-Zr25 condition. It is an ECM protein derived by astrocytes and known to be inhibitory for neurito/axono- (Asher et al., 2000) and synaptogenesis and abundant only in immature synapses (Barros et al., 2011; Pyka et al., 2011). Also the downregulated semaphorin 3 is a long-known repellent for hippocampal axons (Chédotal et al., 1998).

As aforementioned, vesicle transport and membrane trafficking are key events for axonogenesis and many synaptic functions and are strongly affected by the neuron/ns-Zr25 interaction (Figure 5A). The Rab protein family in particular is very prominently involved in these processes and their dysfunction can cause severe neurological disorders (Stenmark, 2009; Villarroel-Campos et al., 2014). In the neurons on ns-Zr25 several Rab proteins were found to be upregulated. Among these Rabs is e.g., Rab3. It is important for hippocampal synaptic plasticity and vesicle priming to optimize synaptic transmission (Schlüter et al., 2006). Rab5 and Rab7, found also in IAC, participate in the fine-tuning of cell adhesion. They reorganize the actin cytoskeleton (Lanzetti et al., 2004), spatiotemporally modulate FA dynamics (Palamidessi et al., 2013) and orchestrate the recycling and trafficking of active and inactive β1 integrins (Arjonen et al., 2012). The upregulated CLIC4 and Arfs are known to contribute to these processes (Norman et al., 1998; Myers and Casanova, 2008; Argenzio et al., 2014). In the neuronal context, both Rab5 and Rab7, regulate the axonal retrograde transport and therewith the neurotrophin and N-Cadherin trafficking (Deinhardt et al., 2006; Kawauchi et al., 2010). Rab5 is furthermore important in evoked neurotransmitter release (Wucherpfennig et al., 2003). It is also congruent that syntenin-1 is upregulated, an adaptor protein with versatile roles involved in neuronal membrane architecture and synapse formation, e.g. by regulating the trafficking of receptors and cell adhesion proteins (Hirbec et al., 2005; Beekman and Coffer, 2008). Remarkably, the only Rab found to be downregulated, Rab31, has been recently shown to be involved in the control of neural progenitor cell (NPC) differentiation and the astrocyte/neuron switch (Chua C. E. L. et al., 2014). Regarding this switch toward neurons, also the upregulation of apoE is quite intriguing. It is essential for lipid homeostasis and receptor-mediated endocytosis of lipid particles and its knockout leads to a reduction of neuro- and augmentation of astrogenesis in hippocampal NPC (Li et al., 2009; Schinder and Morgenstern, 2009). Another important protein associated with vesicle transport and axonal/dendritic outgrowth is the upregulated AP180 (SNAP91). Its overexpression causes the formation of multiple axons in hippocampal neurons whereas its knockout, respectively reduction, impairs axonal/dendritic development (Bushlin et al., 2008) leading to less and smaller synaptic vesicles (Petralia et al., 2013). Furthermore, RanBP is upregulated which is pivotal in the regulation of axonal retrograde signaling to the nucleus (Panayotis et al., 2015).

Another important cellular process is the protein turnover and degradation which in particular for neurons is challenging to manage because of their special morphology and large cell surface. In fact, the wide range of neurodegenerative diseases caused by ubiquitin-positive protein aggregations speaks for itself and pinpoints to this difficulty (Tai and Schuman, 2008). Moreover, the ubiquitin-proteosome system has an eminent function in neuro- and synaptogenesis by the selective and targeted degradation of substrates with fundamental roles in these processes (Tai and Schuman, 2008; Tuoc and Stoykova, 2010). Many components of this system have been found to be altered in the ns-Zr25 condition (Figure 5B), all upregulated. One interesting example with a prominent function in neurons is UBE3A, which can be found in the nucleus, synapses and dendritic spines of hippocampal neurons. It participates in the synaptic development (Dindot et al., 2008) and loss of function mutations in this protein lead to impairment of hippocampal long-term potentiation and the neurological disorder Angelman syndrome (Jiang et al., 1998). Ube2i/UBC9, a protein involved in sumoylation, is instead downregulated. This protein is important for the maintenance of pluripotency in embryonic stem cells (Tahmasebi et al., 2014). A high expression level of this protein has been reported in neural stem cells whereas in differentiated neurons it is only moderately expressed (Watanabe et al., 2008).

On the level of the nucleus and transcriptional control, some interesting proteins are altered. The upregulated RNA-binding protein FUS e.g., has many mRNA targets in the neuronal transcriptome regulating synaptic functions and cell adhesion (Nakaya et al., 2013). This protein can be found in FA and is involved in initial cell spreading events (de Hoog et al., 2004). Another upregulated RNA-binding protein is ELAVL3/HuC which contributes to the control of neurogenesis and neuronal differentiation/maturation (Akamatsu et al., 2005). FRX2 instead is downregulated and known to be a negative regulator of translation (Laggerbauer et al., 2001) with many mRNA targets coding for proteins with neuronal and synaptic functions (Darnell et al., 2001). Also chromatin remodeling is essential for the regulation of gene expression and differentiation, in particular also in the neuronal context (Fischer et al., 2007). Therefore, the observed downregulation of HDAC2 is congruent as HDAC inhibition triggers neurogenesis in NPC (Hsieh et al., 2004) and HDAC2 deficiency promotes synaptic plasticity and neural circuit formation with a positive impact on memory and learning (Guan et al., 2009). The downregulated CREB, which often associates with MeCP2 (Chahrour et al., 2008) (also downregulated), is known to be an essential transcription factor in particular in the critical, earlier GABA-dependent phase of neurogenesis whereas in the later stages of neuronal development and network formation this signaling is downregulated (Jagasia et al., 2009; Pallotto and Deprez, 2014). In PC12 cells grown on neuritogenesis-promoting ns-Zr, we observed an increased nuclear localization of phosphorylated CREB in the beginning which later on decreased (Schulte et al., 2016). In the hippocampal neurons interacting with ns-Zr25 instead, CREB and a GABA_A_ receptor are downregulated which is in line with the more advanced maturation status of the neurons (Jagasia et al., 2009; Pallotto and Deprez, 2014). The contribution of MeCP2 is complex and its expression level and phosphorylation status has to be regulated well to ensure neuronal functions (Chahrour et al., 2008; Cohen et al., 2011; Yao and Jin, 2014). The strongly downregulated HMGB1 has been shown to be downregulated in adult neurons (Guazzi et al., 2003). Interestingly enough, also the transcription factor TSC22D1, which was very recently linked to JNK-dependent (neuronal) differentiation processes (Sahu et al., 2015), is upregulated. From a mechanotransductive point of view also the upregulation of LAP2 (lamina-associated polypeptide 2) is quite interesting, as it is involved in the organization of the nuclear and chromatin structure, and the nucleoplasmic transport of lamin A (Dechat et al., 1998; Osmanagic-Myers et al., 2015), a protein recently found to be essential in mechanotransductive signaling (Swift et al., 2013).

Altogether the proteomic data demonstrates a fundamental change of the cellular program in the hippocampal neurons after 3 days of interaction with the ns-Zr25 compared to the control standard culture condition. The neurons on ns-Zr25 are already at this stage on the course toward mature neurons and in the process of integrating themselves into the forming neural network. Furthermore, this proteomic analysis delivered a first insight into the impact of neuron/nanotopography interaction on prominent components of the mechanotransductive machinery.

The here demonstrated capacity of this biomaterial to affect neuronal development could indeed be very useful for a large variety of biomedical applications, including the development of neurogenesis/neuroinduction-promoting cell culture devices and effective neural interfaces (Kotov et al., 2009; Franze et al., 2013; Mammadov et al., 2013; Fattahi et al., 2014; Tong et al., 2015). The first objective could be instrumental for the creation of *in vitro* models for neurodegenerative diseases, or for the establishment of stem cell-based regenerative cell replacement approaches. Referring to the neural interfaces, it would be important in the near future to test if nanostructured zirconia can be used to design specific neural circuits with predetermined connectivity. As long term challenge it would be interesting to see how these artificial circuits can be integrated in the living brain after implantation. An additional potential outcome of these results is the idea that implanting devices with the nanostructured zirconia surfaces into living neural tissue might reactivate and promote differentiative/maturative programs in animal models of neurodegenerative diseases or spinal cord injuries.

At the present time, these nanotopographical surfaces fabricated by SCBD are a promising tool to further unveil molecular aspects regarding neuronal cell adhesion to extracellular substrates and to comprehend how they regulate and guide neuronal differentiation and maturation, both, in physiological and pathological situations. The impact of biophysical factors on the development of neuronal cells got increasingly appreciated in recent years (Tyler, 2012; Franze et al., 2013; Kerstein et al., 2015) but still requires a more thorough understanding. In this context, the profound proteomic analysis already unraveled several interesting protein candidates of the mechanotransductive signaling pathways for more detailed investigations.

## Author contributions

The project was primarily conceived by CS and MR. CS wrote the principal part of the manuscript. CS, MR, MC, and GT designed and realized the figures. Cell extraction was done by MR. The electrophysiological measurements were done by MR and the corresponding data analyses were performed by MR, JL, and AM. The proteomic experiment and corresponding data analysis was conceived and realized by EM, SN, CS, and GT. Immunofluorescence and corresponding image analysis was performed by MR, MC, and CS. The fabrication of the nanostructured surfaces by SCBD was done by CP. The AFM characterization of the surfaces was performed by LP and AP. PM, AM, GT, CL, and AP were involved in conceiving and creating the project, the realization of the manuscript, furthermore they contributed reagents, materials, and analysis tools.

### Conflict of interest statement

The authors declare that the research was conducted in the absence of any commercial or financial relationships that could be construed as a potential conflict of interest.

## Supplementary material

Tables S1, S2 **Differentially expressed proteins from the proteomic analysis of the comparison between ns-Zr25 and Control**. The tables report the lists of proteins (Table S1) upregulated or expressed only in ns-Zr25, or (Table S2) downregulated in ns-Zr25 or expressed only in Control. The column Razor + unique peptides indicate the number of unique and razor peptides associated to a protein. Mitominer true proteins are marked in light gray. Proteins present in the adhesome list published by Winograd-Katz et al. (2014) are marked in bold in the Gene names column.

Tables S3, S4 **Differentially expressed proteins from the proteomic analysis of the comparison between ns-Zr25 and flat-Zr**. The tables report the lists of proteins (Table S3) upregulated or expressed only in ns-Zr25, or (Table S4) downregulated in ns-Zr25 or expressed only in flat-Zr. The column Razor + unique peptides indicate the number of unique and razor peptides associated to a protein. Mitominer true proteins are marked in light gray. Proteins present in the adhesome list published by Winograd-Katz et al. (2014) are marked in bold in the Gene names column.

Table S5 **IAC proteins [according to Geiger and Zaidel-Bar (2012)] dissected from Tables S1, S2 (for the comparison ns-Zr25 vs. Control) or from the Tables S3, S4 (for the comparison ns-Zr25 vs. flat-Zr)**.

Figure S1 **Proteomic analysis and Gene Annotation enrichment analysis for the comparison between ns-Zr25 and flat-Zr. (A)** Work flow of the proteomic approach. A shotgun proteomic analysis was performed on the hippocampal neurons cultured for 3 days either on flat or on the nanostructured zirconia surface with a roughness R_q_ of 25 nm rms. Statistical analyses were performed using the Perseus software (version 1.4.0.6, www.biochem.mpg.de/mann/tools/). Only proteins present and quantified in at least 3 out of 5 technical repeats were considered as positively identified in a sample and used for statistical analyses. Proteins were considered differentially expressed if they were present only in ns-Zr25 or flat-Zr or showed significant *t*-test difference (cut-off at 5% permutation-based False Discovery Rate). **(B,C)** The Gene annotation enrichment analysis was carried out on proteins upregulated or expressed only in ns-Zr25. The proteins differently expressed were clustered according to their functions using the Panther platform (Version 10.0 release date April 25, 2015) filtered for significant Gene Ontology terms: Biological Process (GO-SlimBP) **(B)** and Pathways **(C)** using a *p* value < 0.05. The fold enrichment value is reported in the y-axis. The numbers in bold above each bar indicates the number of genes enriched in the analysis.

## Abbreviations

AFM: atomic force microscopy
DIV: days *in vitro*
ECM: extracellular matrix
eGFP: enhanced green fluorescence protein
FA: focal adhesion(s)
IAC: integrin adhesion complex
mPSC: miniature postsynaptic currents
NPC: neural progenitor cells
ns-Zr: nanostructured zirconia
pA: pico ampere
PO: polyornithine
rms: root mean square
SCBD: supersonic cluster beam deposition
*SD*: standard deviation
SEM: standard error of the mean
TTX: tetrodotoxin.
