# Supplementary figures and images for: Scale Invariant Disordered Nanotopography Promotes Hippocampal Neuron Development and Maturation with Involvement of Mechanotransductive Pathways
Source: Front Cell Neurosci. 2016 Nov 18;10:267. doi: 10.3389/fncel.2016.00267 (PMC5114288; doi:10.3389/fncel.2016.00267)

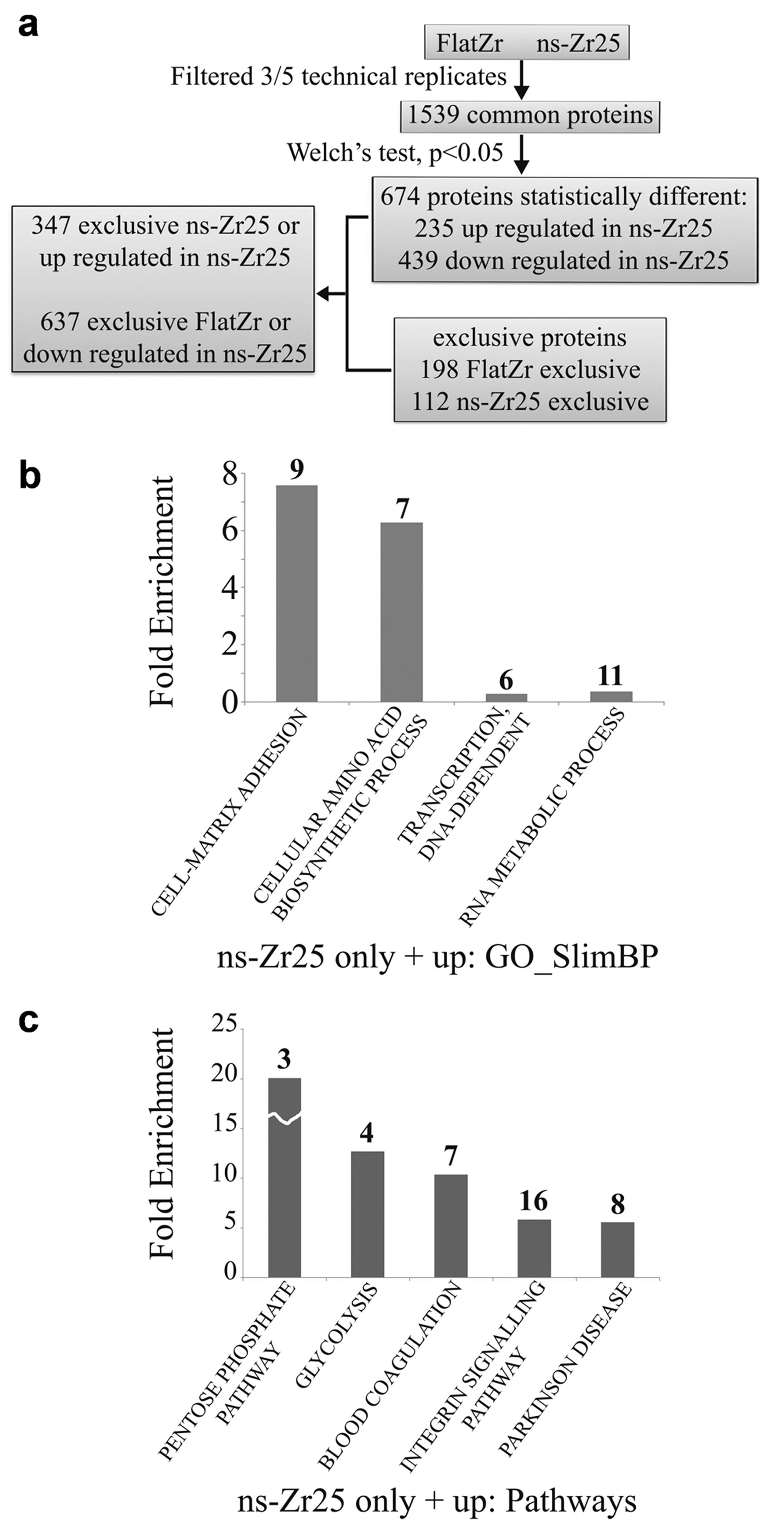

Supplement: Figure S1 — Proteomic analysis and Gene Annotation enrichment analysis for the comparison between ns-Zr25 and flat-Zr. (A) Work flow of the proteomic approach. A shotgun proteomic analysis was performed on the hippocampal neurons cultured for 3 days either on flat or on the nanostructured zirconia surface with a roughness Rq of 25 nm rms. Statistical analyses were performed using the Perseus software (version 1.4.0.6, www.biochem.mpg.de/mann/tools/). Only proteins present and quantified in at least 3 out of 5 technical repeats were considered as positively identified in a sample and used for statistical analyses. Proteins were considered differentially expressed if they were present only in ns-Zr25 or flat-Zr or showed significant t-test difference (cut-off at 5% permutation-based False Discovery Rate). (B,C) The Gene annotation enrichment analysis was carried out on proteins upregulated or expressed only in ns-Zr25. The proteins differently expressed were clustered according to their functions using the Panther platform (Version 10.0 release date April 25, 2015) filtered for significant Gene Ontology terms: Biological Process (GO-SlimBP) (B) and Pathways (C) using a p value < 0.05. The fold enrichment value is reported in the y-axis. The numbers in bold above each bar indicates the number of genes enriched in the analysis. [file Image1.TIF]
